# FANTOM4 EdgeExpressDB: an integrated database of promoters, genes, microRNAs, expression dynamics and regulatory interactions

**DOI:** 10.1186/gb-2009-10-4-r39

**Published:** 2009-04-19

**Authors:** Jessica Severin, Andrew M Waterhouse, Hideya Kawaji, Timo Lassmann, Erik van Nimwegen, Piotr J Balwierz, Michiel JL de Hoon, David A Hume, Piero Carninci, Yoshihide Hayashizaki, Harukazu Suzuki, Carsten O Daub, Alistair RR Forrest

**Affiliations:** 1 RIKEN Omics Science Center, RIKEN Yokohama Institute, 1-7-22 Suehiro-cho Tsurumi-ku Yokohama, Kanagawa, 230-0045 Japan; 2 Biozentrum, University of Basel, and Swiss Institute of Bioinformatics, Klingelbergstrasse, CH-4056 Basel, 4056, Switzerland; 3 The Roslin Institute and Royal (Dick) School of Veterinary Studies, The University of Edinburgh, Roslin, EH259PS, UK; 4 The Eskitis Institute for Cell and Molecular Therapies, Griffith University, Brisbane Innovation Park, Don Young Road, Nathan, QLD 4111, Australia

## Abstract

EdgeExpressDB is a novel database and set of interfaces for interpreting biological networks and comparing large high-throughput expression datasets that requires minimal development for new data types and search patterns. The FANTOM4 EdgeExpress database  summarizes gene expression patterns in the context of alternative promoter structures and regulatory transcription factors and microRNAs using intuitive gene-centric and sub-network views. This is an important resource for gene regulation in acute myeloid leukemia, monocyte/macrophage differentiation and human transcriptional networks.

## Abstract 2

EdgeExpressDB is a novel database and set of interfaces for interpreting biological networks and comparing large high-throughput expression datasets.

## Rationale

The FANTOM4 Expression Cluster Workshop [1] is part of the Genome Network Project [2] and is the next phase of the FANTOM (Functional Annotation of Mammals) project [3-5]. For FANTOM4 the human transcriptional regulatory network was studied in a myeloid leukemia cell line (THP-1) [6] undergoing differentiation induced by phorbol-myristate-acetate. For detailed descriptions of the data collected and analyses used for each of the edge types contained within EdgeExpressDB, we refer the reader to the FANTOM4 main paper [1]; however, here we introduce the data in brief (Additional data file 1). The genome-wide dynamics of transcription start site (TSS) usage along a time course was measured experimentally. This was achieved by adapting cap analysis of gene expression (CAGE) [7] to deepCAGE (deep sequencing on a next generation sequencing platform, in this instance a 454 sequencer). On average, each sample is sequenced to a depth of one million deepCAGE tags, and for this project we mapped a total of 17 million tags to 2.8 million positions. This allowed us to identify the set of promoters active during differentiation, their dynamics and the individual TSS positions used for each. Using the promoter regions defined by deepCAGE and their expression profiles, we predicted the conserved transcription factor binding sites (TFBSs) within these regions most likely to explain the expression of the promoter, using motif activity analysis (described in [1]). In addition to these data, a diverse set of expression measurements and edge types were amassed (microarray expression, chromatin immunoprecipitation (ChIP)-on-chip, small interfering RNA (siRNA) perturbation, and microRNA (miRNA) over-expression, as well as the protein-protein interactions and quantitative real-time PCR (qRT-PCR) expression patterns of transcription factors).

In order to interpret all of these data in the context of a genome-scale regulatory network, miRNA-target and transcription factor-target regulation needs to be analyzed and integrated with transcription factor protein-protein interactions and RNA expression measurements for every component. One of the goals from the outset of the project was to make the predictions, promoters, and expression data easily available to end users. To address this we developed the EdgeExpress database (EEDB) with views of the data that integrate the expression, genomic organization, and regulatory (miRNA, TFBS and protein-protein) edges.

## Access to the FANTOM4 data via EdgeExpressDB

One of our prime goals was to make this high throughput data easily available to end user biologists in an integrated form. We therefore developed both a gene-centric and a sub-network view (Additional data files 2 and 3). The gene-centric view presents the user with a summary of observed promoters, promoter expression, transcription factors known and predicted to regulate the gene as well as the miRNAs that target the transcript. The sub-network query tool (Additional data file 3) allows users to view subsections of the predicted network by providing a list of gene or miRNA symbols. For both of these views we provide a rapid free word search at the top, which updates as each letter of the keyword is entered (for example, as the user types the letters a,b,c, the query returns all (ABC*) ATP-binding cassette protein members; an additional 'a' changes the query to (ABCA*) ATP-binding cassette protein subfamily A members, and so on). While the views primarily focus on Entrez Gene entries [8], and miRbase miRNAs [9], the search system also works on aliases, descriptions, keywords, FANTOM4 promoter identifiers, and microarray probe identifiers.

## Gene-centric view

The gene-centric view was designed to aid biologists who are interested in the regulation of a specific gene. Using the rapid search described above the user can select the gene (or feature) they are interested in. The view is composed of three horizontal panels (with the top panel split into 3 vertical sections; Additional data file 2). This page summarizes the genomic structure of the gene (genome view bottom panel), expression of the gene (biological triplicate time-course measurements by deepCAGE and microarray), regulatory inputs (top left), gene annotation and protein-protein interactions (top middle), and the regulatory targets for transcription factor genes and miRNAs as derived from predictions, literature and perturbation experiments (top right). With this view, all information and interactions pertinent to the gene or miRNA of interest is available for inspection.

A discriminating feature of the FANTOM4 project was its use of deepCAGE to identify active promoters and measure the genome-wide dynamics of TSS usage during differentiation. The gene-centric view provides an integrated overview of the genomic position, expression dynamics and predicted regulators of these promoters. To describe the relationship between TSSs and promoters, we developed the following terminology. Individual TSSs are referred to as level 1 (L1), nearby TSSs whose expression profiles are the same up to measurement noise are clustered into promoters (L2), and adjacent promoters that are within 400 bp of each other are condensed into 'promoter regions' (L3). The gene-centric view displays: the expression of L2 and L3 promoters in the center horizontal panel (and matching microarray or qRT-PCR measurements if available); the position of the promoters relative to the annotated transcripts (bottom panel); and the factors and TFBSs predicted to regulate the expression of the promoter (bottom panel) and a weight on the strength of the prediction (top left panel). This makes it easy for a user to see which promoter is active for a given gene, its expression relative to microarray measurements, and the predicted TFBSs most likely to explain the observed expression. If the user mouses over a transcription factor input, it will show the response weight for that instance of a site. The higher the value, the more likely the L2 promoter is regulated by that factor. For more information on the response weight and motif activity analysis in general, please refer to the FANTOM4 main paper [1]. Note that according to our siRNA perturbation experiments, TFBS predictions with response weights > 1.5 are more likely to validate.

In addition to the FANTOM4 transcription factor-target predictions, the left and right panels also incorporate transcription factor-target edges from: public and in-house ChIP-on-chip experiments (the FANTOM4 PU.1 and SP1 ChIP-on-chip data are also shown in the genome view, bottom panel); published protein-DNA edges; and focused siRNA perturbation experiments. The other edge types shown in this view are miRNA-target predictions from EIMMO [10] and publicly available protein-protein interactions for all human transcription factors. For all published edges we provide links back to their source (generally a PubMed link). Further description of the edges and weights for each type are also provided (Additional data file 4).

Finally, the genome view provided is a conventional genomic view centered on the gene of interest using annotated Entrez Gene or mirBase genomic co-ordinates. The tracks displayed include known transcripts and small RNAs, L2 and L3 promoters, microarray probes, TFBS predictions and ChIP-chip signal for PU.1, SP1, and acetylated H3K9 and enable users to relate CAGE signal to alternative promoters and transcript isoforms [11]. To access any of these tracks in further detail, the image is hyperlinked back to the corresponding region in the FANTOM4 genome browser, which is based on the generic genome browser [12]. In addition, for users interested in extracting individual promoter regions or TFBS instances, clicking on the L3 promoters in the input region will launch a genome browser window centered on the promoter and the (-300 bp, +100 bp) region used for TFBS predictions. From here users can export GFF format files, or sequence using Gbrowse. Conversely, we provide links back to features in EEDB from the genome browser.

## Sub-network view

Often researchers are interested in the regulatory interactions between a group of genes and miRNAs. For example, given a set of candidate genes (for example, genes mutated in leukemia or co-regulated in a microarray experiment), what are the predicted edges between them and which of these have experimental support? We therefore developed a sub-network search tool (Additional data file 3) that, given a set of genes/miRNAs and a users selection of edge type, will search for all matching connecting edges between those genes and use Graphviz [13,14] to draw an SVG image (scalable vector graphics format) of the resulting sub-network for all nodes with at least one connection.

To begin users need to provide a list of identifiers to be pasted into the text box provided or add them step-wise from sets of genes returned from the rapid query box at the top of the page. If the user then hits the 'SVG preview' button they will be presented with a graphical view of the known and predicted regulatory edges between these nodes. This is the simplest query and returns a network graph showing all edges in the database between any two of the nodes. The diameter of each node is scaled to indicate the 'dynamics' of the gene (based upon Illumina microarray expression measurements) and the color is used to reflect the expression at the currently selected time-point. This allows users to see which network components are co-expressed and how the expression of interconnected nodes changes during a time-course. In addition, the nodes are hyperlinked back to the gene-centric view for more details on a particular feature.

For the edges, the 'edge type' is represented by different colors, the 'edge weight' is represented by the thickness of the line, and 'inhibitory', 'activating' and 'non-directional' edges are represented by lines with flat, pointed or no arrowheads, respectively. Users have control over which edge types are shown and can also make more complex queries to find pairs of nodes connected with multiple lines of evidence. For example, this is useful for viewing which predicted interactions have independent experimental support from ChIP-chip, perturbations or the published literature. In addition, users can trim or expand the currently displayed sub-network as desired using the 'hide singletons', and 'hide leaves' buttons.

Finally, the resulting networks can be exported as SVG image files for publication purposes and as several other output formats, including the cytoscape [15] compatible SIF format, EEDB custom 'xml' format and a simple 'subnet gene list' of nodes remaining from the search.

## A unique resource for gene regulation and acute myeloid leukemia

EEDB integrates a unique combination of predictions and high-throughput experimental data for a human transcriptional network undergoing differentiation. It is particularly relevant to researchers interested in differentiation of the myeloid lineage and acute myeloid leukemia, but also provides regulatory information for most human genes.

In the THP-1 model (an M5 monoblast like acute myeloid leukemia), we carried out systematic knock-down followed by expression profiling for a collection of 52 transcription factors (BCL6, BMI1, CBFB, CEBPA, CEBPB, CEBPD, CEBPG, CTCF, E2F1, EGR1, ETS1, ETS2, FLI1, FOXD1, FOXJ3, FOXP1, GATA2, GFI1, HOXA9, HOXA10, HOXA11, HOXA13, ID1, IRF7, IRF8, IRX3, LMO2, MAFB, MLL, MLLT3, MXI1, MYB, MYBL2, MYC, NFE2L1, NFKB1, NFYA, NOTCH1, NRAS, PTTG1, RUNX1, SNAI1, SNAI3, SP1, SPI1(PU.1), SREBF1, STAT1, TCFL5, TRIM28, UHRF1, YY1, ZNF238). Many of these play key roles in myeloid differentiation [16,17] or have been implicated in acute myeloid leukemia [18,19]. The siRNA experiments and TFBS predictions allow researchers to examine sets of predicted direct and indirect targets of these transcription factors.

EEDB also provides users with a more integrated view of how individual genes are regulated, both at the level of alternative promoter structure and as part of a network (for an example focused on the prototypic monocytic marker CD14, see Additional data file 5).

## Data abstraction

To integrate such a variety of data types and analysis in a single framework, we adopted a snow-flake schema design [20] to model biological data as three major concepts: features, edges, and expression (Figure 1). The flexibility of these generic abstractions allowed all FANTOM4 data to be loaded into the database, and the simple design provided fast searching and data access. A summary of the features, edges and expression measurements provided in the FANTOM4 instance of EEDB is shown in Tables 1, 2 and 3 and the abstractions described below.

**Figure 1:**
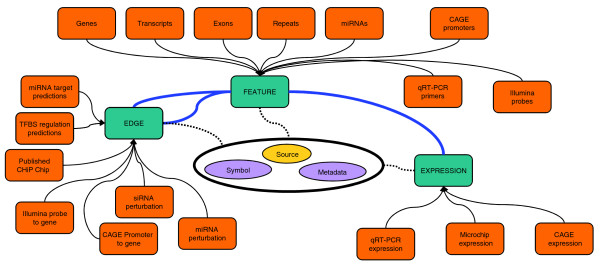
EdgeExpressDB design and data abstraction. EdgeExpressDB is based on three core concepts: feature, edge and expression. Note the two way connection of edges to features and that for each of these elements metadata containing the symbol and source can be provided. This allows for all data from the FANTOM4 project (represented by orange boxes) to be mapped into the system.

**Table 1:** Contents of the FANTOM4 instance of EdgeExpressDB: features

Features	Source	Genomic co-ordinates
Gene	Entrez [8]	Yes
Transcription start site (L1)	FANTOM4 [1]	Yes
Promoter (L2)	FANTOM4 [1]	Yes
Promoter (L3)	FANTOM4 [1]	Yes
miRNA (pre-)	Mirbase 10.0 [9]	Yes
miRNA (mature)	Mirbase 10.0 [9]	No
Illumina probe	FANTOM4 [1]	Yes/no
Agilent miRNA probe	FANTOM4 [1]	No
TFBS matrix	FANTOM4 [1]	No

**Table 2:** Contents of the FANTOM4 instance of EdgeExpressDB: edges

Edges	Source	Relationship
TF-promoter (L2)	Predictions (FANTOM4 [1])	Belongs to
Promoter (L2)-promoter region (L3)	FANTOM4 [1]	Belongs to
Promoter region (L3)-gene	FANTOM4 [1]	Belongs to
Promoter region (L3)-pre-miRNA	FANTOM4 [1]	Belongs to
Pre-miRNA-mature miRNA	FANTOM4 [1]	Belongs to
miRNA-target	ElMMo (SIB) [10]	Interaction
Protein-protein	Published	Interaction
TF-gene	Published	Interaction
TF-gene	ChIP-chip	Interaction
TF-gene (perturbation edge)	siRNA knockdown	Interaction
miRNA-target (perturbation edge)	miRNA overexpression	Interaction

TF, transcription factor.

**Table 3:** Contents of the FANTOM4 instance of EdgeExpressDB

Expression	Source	Samples
CAGE-L2/L3-gene	FANTOM4 [1]	THP-1 PMA
Illumina microarray-gene	FANTOM4 [1]	THP-1 PMA, siRNA and miRNA perturbations
Agilent microarray-mature miRNA	FANTOM4 [1]	THP-1 PMA

PMA, phorbol-myristate-acetate.

A feature is generally a genomic object (for example, gene, exon, promoter, CAGE tag) with a name and a set of co-ordinates for a particular genome build (for example, chr1 12345670 12345690 + Hg18). However, features do not require co-ordinates and other data types, such as mature miRNAs, qRT-PCR primer sets and unmapped microarray probes, can thus be stored in this system.

An edge is loosely defined as a connection between two of the above features. Edges can have a direction (A regulates B versus B regulates A) and a weight. Weights allow the strength or trust value to be attached to an edge, and a negative value discriminates inhibitory interactions from activating ones. In EEDB, edges are used both in the context of biological interactions (for example, transcription factor A interacts with promoter of gene B; or protein A binds protein B) and for handling belongs-to relationships (that is, promoter 1 belongs to gene B, exon 1 is part of transcript X).

Expression is a measurement on a feature, with raw and normalized expression values and a detection score for a particular experiment. In the case of microarray measurements for a particular gene, we separate expression on a probe from the mapping of the probe to a particular gene (that is, expression to probe to gene). This allows probe mappings to be updated independently of the expression associated to it and also allows for probes that map to multiple loci.

Each of these elements (feature, edge, and expression) is associated with a data source. All elements and sources can be annotated with metadata managed in a unified sub-system.

## Implementation

**Figure 2:**
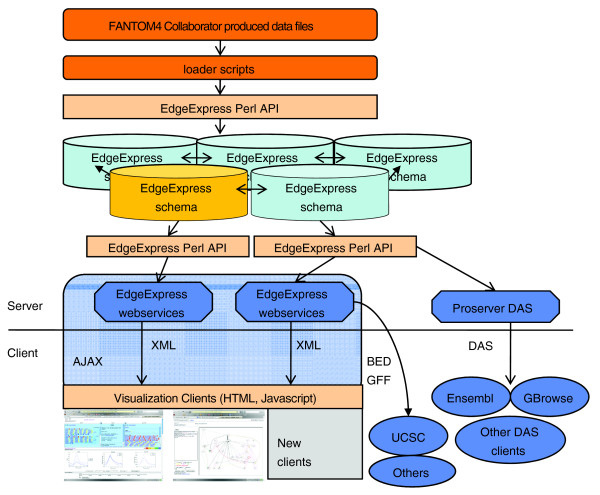
Overview of EdgeExpressDB, federation, web-services and clients. Using loader scripts that communicate through the EdgeExpress perl API, the features, edges and expression are loaded into an instance of the EEDB schema. Multiple instances of EEDB can communicate in a federation through the perl API. The EdgeExpress webservices export data in XML, BED, and GFF3 formats directly and DAS through ProServer integration, which allows AJAX clients and genome browsers to access the data.

To build the views and search systems, we used Web2.0 AJAX technology to provide a more interactive website and to provide multi-purpose data servers. The backend database system was built using perl and mysql. To facilitate development, the EdgeExpress object API toolkit was created as the foundation of the system. This toolkit provided flexibility in developing loader scripts for multiple data types and was also used for the server solutions (Figure 2). The EEDB perl object API layer not only provides for easy development, but also provides an object caching system to enhance performance of the scripts and server solutions. The system was also designed to be fully federated. Although this is currently not needed for the FANTOM4 instance, the federation will allow us to easily expand the data integration and compare FANTOM4 data to other datasets in the future.

By applying AJAX techniques, we were able to keep many aspects of data visualization on the client side with minimal impact on the server side. This allowed us to not only rapidly modify the 'Look and feel' of the system, but also allowed us to add features to the server side solutions in parallel. One aspect of EEDB is that it was first deployed as a 'collaborator' visualization website. As the FANTOM4 project progressed, new datasets became available and were loaded into the 'live' system. Using EEDB these became immediately visible on the websites without needing any system restarts or 'rebuilds'. When working with so many different and large data sets, the ability to append data into the integrated database was a critical feature of the system and for the FANTOM4 collaboration process.

The XML web-services driving the JavaScript interfaces can also be used directly [21]. In addition to XML access to features, edges, expression, and networks, this web-service can also provide the data in dynamic 'genomic region' queries in GFF and BED formats. The FANTOM4 EEDB also provides DAS server support [22] for all genomic mapped features through ProServer [23] integration with the EdgeExpress perl API.

Finally, at the time of writing this paper, the FANTOM4 EEDB contained over 102.1 million rows (10.85 million features, 6.12 million edges, 51.73 million expression points and 33.4 million metadata rows). We currently have three other instances of EEDB containing an additional 456.65 million rows (346.76 million, 53.20 million, and 56.69 million). We have also tested the system with an instance containing 1.959 billion rows and 239 Gigabytes. With the federation, the EEDB system is scalable, and as more large datasets become available more EEDB instances can be established and inter-connected.

## Comparison to other resources

For comparison to other resources, we first compare the FANTOM4 instance of EEDB and the data contained within to similar genomic resources, and then compare the EEDB system to other pre-existing systems.

The FANTOM4 instance of EEDB contains a unique combination of dynamic TSS usage, expression weighted TFBS predictions, microarray expression, siRNA perturbation experiments and transcription factor protein-protein interactions. The majority of these data are not available in an integrated form from any other source. For the promoter annotation we can draw similarities to resources such as MPromDb [24], ORegAnno [25] and EDGEdb [26] that catalog protein-DNA edges for various organisms, and our own CAGE basic and analysis databases [27] established for displaying the CAGE data from FANTOM3. Similarly, there are several more extensively annotated gene-centric databases, such as the Human Protein Reference Database [28], BioGRID [29], and Genecards [30]. However, none of these combine the depth and combination of data, or the views available in the EEDB gene-centric interface. The closest comparative resource for promoter annotation is DBTSS [31], which in a recent update contained 19 million uniquely mapped 5' ends from multiple species and includes TFBS predictions. However, this resource uses different views, different samples, and does not provide expression-weighted TFBS predictions.

In addition, for FANTOM4 we provide a simple sub-network visualization absent from the above resources. Although tools such as Cytoscape [15], BioLayout [32], STRING [33] and the commercial package Ingenuity Pathway Analysis [34] may provide greater functionality for these graphs, to our knowledge no currently available tool provides the combined features of the EEDB package and the novel data content.

Finally, the closest relatives of the EEDB system are Biomart [35] and Ensembl Compara [36]. The main difference is that EEDB is designed to be a generic system for large systems biology datasets (features, networks and expression) implemented as a federated and scalable solution that allows for live updates of existing databases. In contrast, BioMart is essentially a feature-metadata system with no inherent support of networks or expression data searching. Also, the Biomart MartBuilder tool needs to build a new 'mart' when new data are added to the system, which can take weeks to complete when building large marts such as the Ensembl biomart. EEDB can append data into existing databases, and at a rate of 19 million rows per hour per federated database instance.

While Ensembl Compara is a monolithic connection database focused on inter-species gene families, gene evolution, and genomic conservation, EEDB is a generic system for comparing and connecting any type of OMICS data (the combined fields of genomics, transcriptomics, and proteomics) within a peer-to-peer federation, with interspecies connections just being one type.

## Discussion/future directions

The move towards systems biology and OMICS-based sciences imply an increasing need for storing large amounts of data from diverse sources and comparing them in an integrated fashion. In particular, very large deep sequencing datasets are now being generated to investigate short RNAs [37], protein-DNA interactions [38], transcript isoforms [39], RNA degradation [40] and nucleosome positioning [41]. The EEDB system is a scalable solution to handle these large datasets (tested on billions of rows), and is specifically designed for systems biology datasets (networks and expression). Technically, EEDB enables complex searching with speeds appropriate for websites (seconds not minutes), flexibility for loading new data types into a live system, and rapid development of clients. In addition, as the system is federated we are beginning to integrate publication, protein and public expression data into multiple EEDB servers. Federation also means that EEDB can run parallel queries, do parallel loads into multiple EEDB instances, and can effectively provide unlimited data storage and management.

In this paper we describe two of the current clients, but several others are in development and further custom AJAX clients are encouraged through the provision of fast XML servers. We also make the data readily available to the genomic community through DAS, BED and GFF servers. To encourage further instances of EEDB, the schema, perl code object API toolkit and JavaScript clients are open source and available both on the main website and via CPAN [42]. Since the system was designed to be generic for all OMIC style data, we hope EEDB will be useful for other projects.

Finally, in the context of FANTOM4 and the RIKEN OMICS sciences center, we will continue to generate datasets in this field, and continue to integrate regulatory edge and expression information. We believe EEDB will be an important tool for scalable storage and interpretation of these data. We will also continue to release novel datasets via the FANTOM4 EEDB system as soon as the accompanying papers are released. Soon to be released data include miRNA expression profiles, additional perturbation experiments and novel mammalian two hybrid protein-protein interaction data.

## Abbreviations

API: application programming interface; CAGE: cap analysis of gene expression; ChIP: chromatin immunoprecipitation; EEDB: EdgeExpress database; FANTOM: Functional Annotation of Mouse/Mammals; miRNA: microRNA; qRT-PCR: quantitative real-time PCR; siRNA: small interfering RNA; TFBS: transcription factor binding site; TSS: transcription start site.

## Authors' contributions

JMS designed and implemented the database, the perl API toolkit, the web services, and the sub-network search. AW designed and implemented the gene-centric view. HK provided the generic genome browser implementation. TL provided the miRNA edges. MH implemented the expression graphs. EVN and PB provided the promoter clusters and TFBS predictions. PC designed and supervised the CAGE libraries preparation. DH, YH, HS, and CD were involved in conceptualization and supervision. ARRF conceived the views and compiled the data. ARRF and JMS wrote the manuscript.

## Additional data files

The following additional data are available with the online version of this paper: a document that summarizes the current data stored in EEDB at the time of publication and provides the accession numbers for each of the raw data sets (from CIBEX and DDBJ) (Additional data file 1); a PDF showing the *EGR1 *gene as an example in the gene centric view of EEDB (Additional data file 2); a PDF showing the sub-network view of EEDB (Additional data file 3); a document showing the information available as popups in EEDB (edge types and edge weights used in EEDB, CAGE defined promoters, and an explanation of the subnet view) (Additional data file 4); a PDF showing an example of how EEDB can be used with gene-centric and sub-network views for the key monocytic marker CD14 (Additional data file 5).

## Supplementary Material

Additional File 1 Summary of the current data stored in EEDB at the time of publication, and the accession numbers for each of the raw data sets (from CIBEX and DDBJ). Click here for file

Additional File 2 The *EGR1 *gene as an example in the gene centric view of EEDB. Click here for file

Additional File 3 The sub-network view of EEDB. Click here for file

Additional File 4 Information available as popups in EEDB (edge types and edge weights used in EEDB, CAGE defined promoters, and an explanation of the subnet view). Click here for file

Additional File 5 An example of how EEDB can be used with gene-centric and sub-network views for the key monocytic marker CD14. Click here for file
