# Supplementary material for: FANTOM4 EdgeExpressDB: an integrated database of promoters, genes, microRNAs, expression dynamics and regulatory interactions
Source: Genome Biol. 2009 Apr 19;10(4):R39. doi: 10.1186/gb-2009-10-4-r39 (PMC2688930; doi:10.1186/gb-2009-10-4-r39)
Supplement: Additional File 1 — Summary of the current data stored in EEDB at the time of publication, and the accession numbers for each of the raw data sets (from CIBEX and DDBJ). [file gb-2009-10-4-r39-S1.doc]

**Additional data file 1**

This supplementary note gives a brief introduction to the data stored in EEDB at the time of manuscript preparation (Note: extra data will be added in the near future – please see the data release tab from EEDB). It also provides the accession numbers for the raw CAGE sequences stored at DDBJ [43] and the microarray expression data stored in MIAME compliant format at CIBEX [44]. For detailed descriptions of the FANTOM4 project, CAGE data generation and the Motif Analysis Response Analysis (MARA) used to make expression weight Transcription factor binding site predictions, please refer to the FANTOM4 main paper [1].

Biological samples used.

For the FANTOM4 project we studied a time-course of phorbol myristate acetate induced differentiation of the M5 acute myeloid leukemia (AML) cell line THP-1. The differentiation was carried out as biological triplicate time-courses. The RNA samples prepared from these were measured using Illumina whole genome microarrays (Human Sentrix 6 version 2), and Cap Analysis of Gene Expression (CAGE [7]), and the expression patterns of each of the replicate time-courses is displayed in the EEDB expression panel (middle).

Genome wide Promoter identification and expression.

Cap Analysis of Gene Expression (CAGE [7]), was adapted to the 454 Life sciences next generation sequencing platform to provide deep-CAGE. The raw CAGE data is available from DDBJ [43] (accession range shown in brackets).

Biological replicate time-course RIKEN1(0h: [DDBJ:AFAAF0000001-AFAAF0513982], 1h: [DDBJ:AFAAA0000001-AFAAA0543260], 4h: [DDBJ:AFAAB0000001-AFAAB0191814], 12h: [DDBJ:AFAAC0000001-AFAAC0474745], 24h: [DDBJ:AFAAD0000001-AFAAD0353461], 96h: [DDBJ:AFAAE0000001-AFAAE0270208]), RIKEN3 (0h: [DDBJ:AFAAG0000001-AFAAG0349448], 1h: [DDBJ:AFAAH0000001-AFAAH0425524], 4h: [DDBJ:AFAAI0000001-AFAAI0358861], 12h: [DDBJ:AFAAJ0000001-AFAAJ0285303], 24h: [DDBJ:AFAAK0000001-AFAAK0327328], 96h: [DDBJ:AFAAL0000001-AFAAL0456381]), RIKEN6 (0h: AFAAM0000001-AFAAM0436519, 1h: AFAAN0000001-AFAAN0499442, 4h: [DDBJ:AFAAO0000001-AFAAO0682996], 12h: [DDBJ:AFAAP0000001-AFAAP0716451], 24h: [DDBJ:AFAAQ0000001-AFAAQ0546880], 96h: [DDBJ:AFAAR0000001-AFAAR0391757]).

CAGE tags were mapped to the genome using an in-house alignment program (Nexalign – Timo Lassmann in preparation). Tag counts for sequences that mapped to multiple locations were split between the locations using a multi-map rescue strategy that uses the mapped tag density around each multi-mapped location to determine weighting of counts to each position [45].

Mapped tags were clustered into promoters and promoter regions, and transcription factor binding sites (TFBS) were predicted using Motevo [1] in the -300 to +100 or each promoter region.

Motif Activity Response Analysis (MARA [1]) was used to calculate a response weight for each promoter with an instance of a TFBS in comparison to the motif activity (a function of all promoters with the given TFBS). This is fully described in detail in the FANTOM4 main paper [1].

Illumina microarray data

Illumina sentrix6 v2 arrays were used for all time-course and siRNA experiments. For the triplicate time-courses ten time-points were profiled (0,1,2,4,6,12,24,48,72,96 hours – [CIBEX: CBX46]).

For siRNA experiments, THP-1 cells were knocked-down with stealth siRNAs against 52 different transcription factors (BCL6, BMI1, CBFB, CEBPA, CEBPB, CEBPD, CEBPG, CTCF, E2F1, EGR1, ETS1, ETS2, FLI1, FOXD1, FOXJ3, FOXP1, GATA2, GFI1, HOXA9, HOXA10, HOXA11, HOXA13, ID1, IRF7, IRF8, IRX3, LMO2, MAFB, MLL, MLLT3, MXI1, MYB, MYBL2, MYC, NFE2L1, NFKB1, NFYA, NOTCH1, NRAS, PTTG1, RUNX1, SNAI1, SNAI3, SP1, SPI1(PU.1), SREBF1, STAT1, TCFL5, TRIM28, UHRF1, YY1, ZNF238). Stealth siRNAs from Invitrogen were transfected for 48hrs, RNA was harvested, and the effects measured relative to a scrambled negative control siRNA transfection. All siRNA experiments were done in biological triplicate and the data is available through CIBEX [CIBEX: CBX47]).

Perturbation edges displayed in EEDB, are identified using LIMMA in the Bioconductor package of R. Quantile normalization of Illumina data and B-statistic calculations were carried out using the lumi and limma packages of Bioconductor in the R statistical language [46-49]. For differential gene expression during the timecourse and between siRNAs and negative control transfections we required a B-statistic ≥ 2.5, fold change ≥ 2 and the gene had to be detected in one of the conditions (average detection score ≤ 0.01).

Chromatin Immunoprecipitation data.

FANTOM4 generated ChIP-chip datasets for RNA polymerase II [CIBEX:CBX44], acetylated histone H3K9 [CIBEX:CBX48], and the transcription factors SPI1(Pu.1) and SP1 [CIBEX:CBX43]. Public ChIP on chip data were extracted for SRF [49], E2F4, ELF1, ETS1, GABPA, RUNX1 [50], YY1 [51], E2F1, E2F4, E2F6 [52], and MYC [53]. Due to the diverse sources of ChIP data and the methods and thresholds used, we took all targets reported by the respective papers and converted them into an Entrez geneID for loading as a ChIP edge in EEDB. Links to the articles in PUBMEDID are provided for all public ChIP data.

Transcription factor Protein-Protein edges.

The protein-protein interactions currently displayed in EEDB are a curated set of transcription factor – transcription factor interactions extracted from a number of public protein-protein interaction sources including DIP [54], BIND [55] and HPRD [28] (which is shown in the source). Only TF-TF interactions from non-high-throughput methods are shown here.
