# Supplementary material for: FANTOM4 EdgeExpressDB: an integrated database of promoters, genes, microRNAs, expression dynamics and regulatory interactions
Source: Genome Biol. 2009 Apr 19;10(4):R39. doi: 10.1186/gb-2009-10-4-r39 (PMC2688930; doi:10.1186/gb-2009-10-4-r39)

Additional data file 2: Gene-centric view of Egr1.

The gene-centric view consists of a free word search at the top which returns a list of feature symbols. Clicking on one of these symbols returns the view below. The view consists of three horizontal panels. The top panel is split into three sections and consists of input -> node -> output. The middle panel shows the expression view for the locus (in this case both CAGE and Illumina microarray data are shown). The lower most panel shows the locus in the context of the FANTOM4 genome browser, and displays the location of transcripts, CAGE promoters, promoter regions and Illumina microarray probes.

FANTOM4 GNP-ECW

RIKEN OSC

Home | Genome Browser | EdgeExpressDB | Data | Paper

FANTOM4 EdgeExpressDB   about   center view   subnet view   contents

Search:

EGR1

EGR1 NAB1 NAB2 TOE1

INPUT → NODE → OUTPUT

INPUT

Sort By: Name Weight

TFBS predictions — Promoters

HNF4A NR2F1 NR2F2 ZEB1
NFE2L1 MAZ RXRG RXRB
RXRA ZEB1 SP1 SNAI3 SNAI1
SNAI2

EGR1 L2.1 — P1 L3

EVN212 matrix EVN115 matrix SRF
GABPB2 ELK1 GABPA ELK4
EVN111 matrix MZF1 ELF4
ELF1 ELF2 PAX5 MAZ PATZ1
TFAP2C TFAP2A

EGR1 L2.2

SRF EVN212 matrix EVN115 matrix
CREB3 ATF5 EVN111 matrix ATF6
GABPB2 ELK1 GABPA ELK4
MEF2C MEF2A MEF2D SP1
MZF1 MAZ ELF4 ELF1 ELF2
JUND JUN JUNB PATZ1
TFAP2C TFAP2A

EGR1 L2.3

EVN212 matrix CREB3 ATF5

NODE

EGR1

Entrez_gene

early growth response 1 [Homo sapiens]

Alias: AT225, G0S30, KROX-24, NGFI-A, TIS8, ZIF-268, ZNF225

chr5:137829080..137832903 (+)
View in FantomDB UCSC
Entrez_gene: (1958)
GeneticLoc: 5q31.1
OMIM: 128990
subnet: internal

Protein-Protein Interactions

CEBPB CEBPB CEBPB CREBBP CREBBP
EP300 EP300 JUN JUN NAB1 NAB1

OUTPUT

Sort By: Name Weight

Perturbation

TGM4 PDGFRB SIM1 CREB3L3
TMEM64 CHPF FUZ KHDRBS3 RNF19A
PDYN ANKRD55 LOC399888 PNMA6A
GPA33 LDHD EBF3 ZBTB16 YTHDC1
BRMS1L LOC653198 C6orf204
LOC645332 BDH1 FAM125B
LOC221442 ELOVL3 TCF15 AQP11
JMJD5 OSTalpha MFSD9 ST7OT1
AFF2 KATNAL2 C8G OGDHL VASH2
SIX2 NFAT5 TSKS COL6A1 OTX1
THBS4 USP32 LASS1 CD19 TRIM14
PPT2 KAZALD1 PACSIN3 CHSY3
CLSTN2 C13orf15 AHRR ZNF763
C11orf45 RGS9BP GRIN3B MPO
NUDT16P C9orf93 NAT6 LOC145783
ECHDC3 LOC162073 APOA1 ZNF69
CYP46A1 GLT25D2 KPNA5 RNF19A
C21orf30 C6orf52 SLC25A35 INF2

Save Source XML File   Save Expression XML File   Key >2 >1.5 >-1.5 <-1.5 <-2

Expression Graph

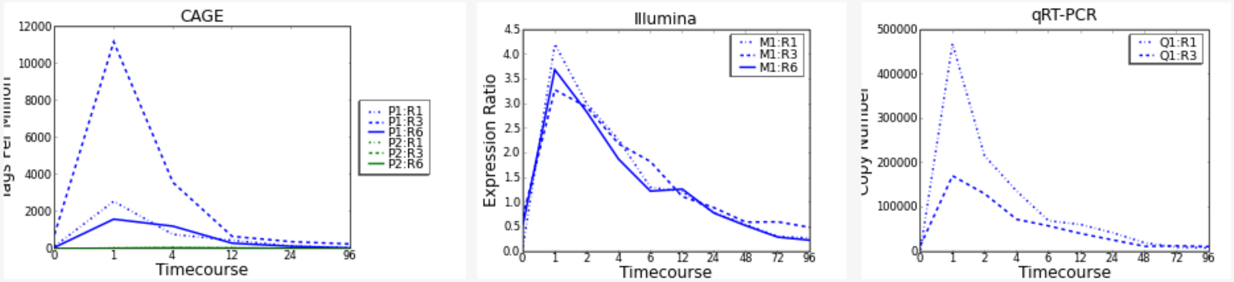

CAGE Promoters (L2 ○ L3 ◉)
L3_chr5_+_137828460[P1] ☑
L3_chr5_+_137829557[P2] ☑

Riken Dataset: 1 ☑ 3 ☑ 6 ☑   Show Data ☐   Reset Graphs

Genome Browser

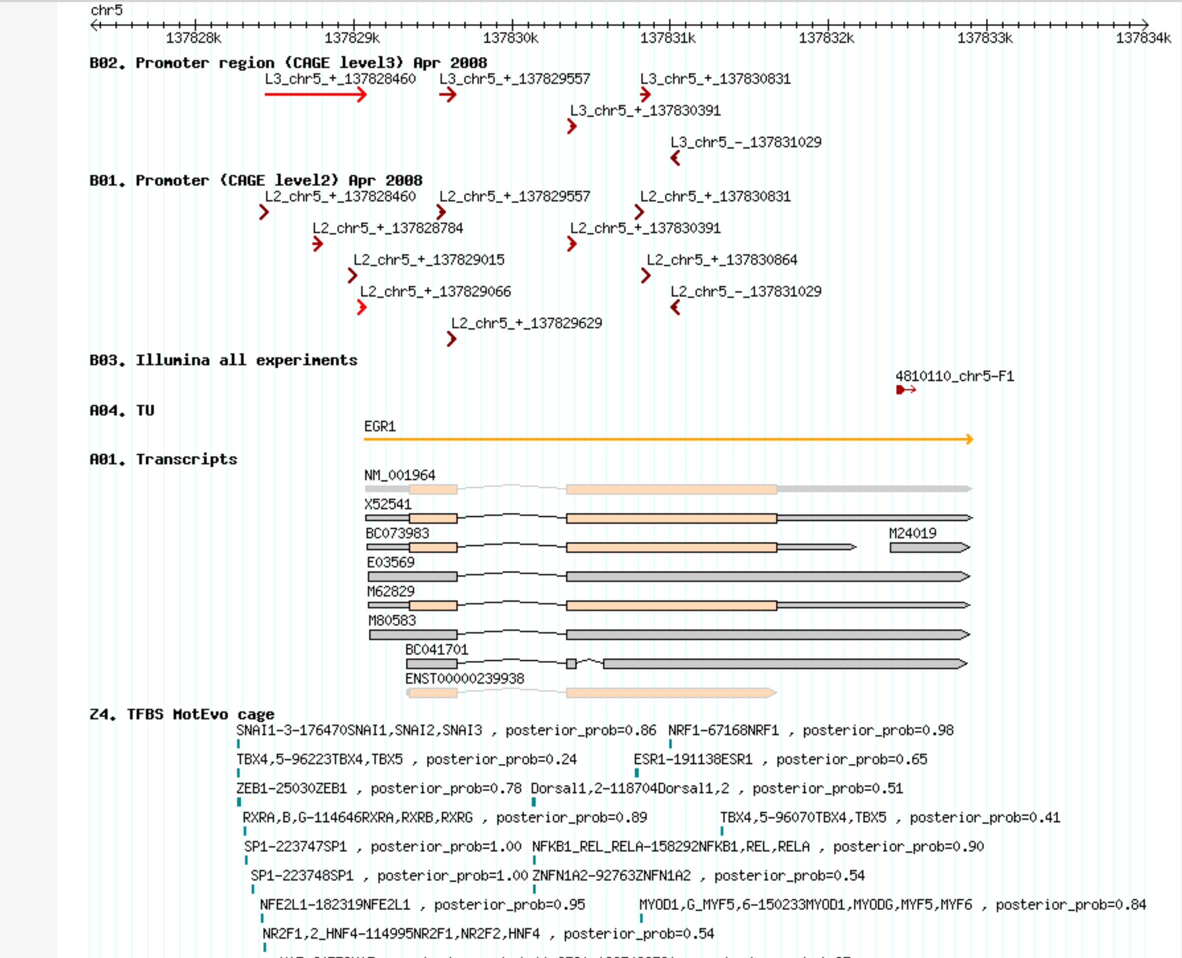

Supplement: Additional File 2 — The EGR1 gene as an example in the gene centric view of EEDB. [file gb-2009-10-4-r39-S2.pdf]
