# Supplementary material for: FANTOM4 EdgeExpressDB: an integrated database of promoters, genes, microRNAs, expression dynamics and regulatory interactions
Source: Genome Biol. 2009 Apr 19;10(4):R39. doi: 10.1186/gb-2009-10-4-r39 (PMC2688930; doi:10.1186/gb-2009-10-4-r39)

Additional data file 3: Sub-network view

The subnet view allows for one to input a list of gene and/or miRNA names as nodes in a graph. The system will search for all matching connecting edges within that set of nodes based on user selectable edge filters. The system allows for simple logic by providing two edge sets (primary and secondary). If only edges in set1 are selected then a simple search is performed. But if edges from both sets are selected the search is performed as ((any edge in set1) and (any edge in set2)) thus requiring two edges to connect node pairs. The available edges are TFBS prediction (black), miRNA target predictions (black), published edges (yellow), PPI edges (purple), ChIP/chip edges (green), and perturbation siRNA/miRNA (red) edges. Round nodes are genes, while hexagonal nodes are miRNAs. The diameter of each node is scaled to indicate the 'dynamics' of the gene by mapping to log(max(detected ILMN expression)/min(detected ILMN expression)) within the time course. The color of the node is mapped to a relative scale for each node between white for min(detected ILMN expression) and purple max(detected ILMN expression). If the node has no detectable ILMN expression, the name of the node becomes red and the background is white.

FANTOM4 GNP-ECW

RIKEN RIKEN OSC

Home | Genome Browser | EdgeExpressDB | Data | Paper

FANTOM4 EdgeExpressDB | about | center view | subnet view | contents

Search:

please enter search term

EEDB subnet LEGEND info

Clear | load genelist cookie

demos: net1 net2 net3 net4 net5

BCL6 EGR1 ETS1 ETS2 FOXD1
FOXP1 FOXP2 GAS6 GRB2 IRF7
KLF10 KLF2 LMO2 MXI1 NAB2
NFAT5 NFE2L1 NFKB1 NRAS RUNX1
SNAI1 SREBF1 SRF TGFBI
TGFBRAP1 TNFAIP3 TNFRSF12A
TNFSF14 TP53INP1 TP53INP2 YY1

primary edge types

- [x] TFBS pred
- [x] miRNA target pred
- [ ] Published protein-DNA
- [ ] PPI
- [ ] ChIP
- [ ] perturbation siRNA/miRNA

AND secondary edge types

- [ ] TFBS pred
- [ ] miRNA target pred
- [x] Published protein-DNA
- [ ] PPI
- [x] ChIP
- [x] perturbation siRNA/miRNA

- [x] hide singletons
- [ ] hide leaves

expression timepoint: 0hr

SVG

generate subnet | SVG preview

IE requires plugin for SVG
please download Adobe SVG plugin

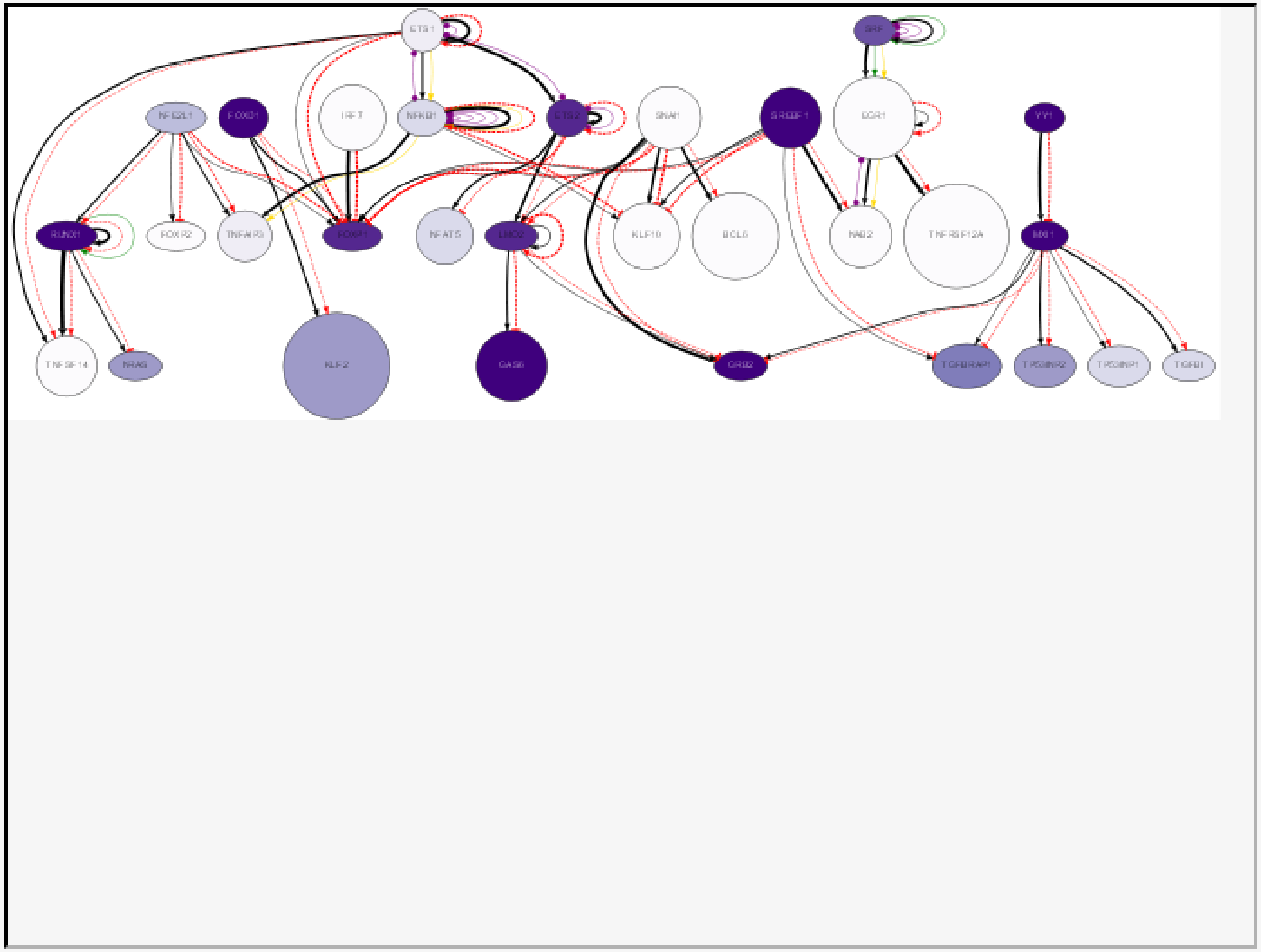

Supplement: Additional File 3 — The sub-network view of EEDB. [file gb-2009-10-4-r39-S3.pdf]
