# Supplementary material for: FANTOM4 EdgeExpressDB: an integrated database of promoters, genes, microRNAs, expression dynamics and regulatory interactions
Source: Genome Biol. 2009 Apr 19;10(4):R39. doi: 10.1186/gb-2009-10-4-r39 (PMC2688930; doi:10.1186/gb-2009-10-4-r39)
Supplement: Additional File 4 — Information available as popups in EEDB (edge types and edge weights used in EEDB, CAGE defined promoters, and an explanation of the subnet view). [file gb-2009-10-4-r39-S4.doc]

**Additional Data file 4**

This supplementary note covers three sets of information available as popups in EEDB.

1. Edge types and edge weights used in EdgeExpressDB.

**TFBS predictions**

**TFBS EDGES:** Evolutionarily conserved Transcription factor binding sites are predicted using MOTEVO with a set of non-redundant matrices (combining JASPAR, TRANSFAC and a small set of de-novo motifs trained on ChIP-chip datasets).

**TFBS WEIGHTS:** The weights on TFBS edges are “response values”, these are central to the FANTOM4 analysis and basically say how well the expression of each LEVEL2 promoter responds to (or matches) the motif activity for that factor (eg. MYB motif activity decreases as the cells differentiate and PRTN3 a known (and predicted) target of MYB is down-regulated, hence it has a high response weight of 14.125). We recommend to users wishing to validate sites, response weights >1.5 are more reliable. For more detail on TFBS prediction and motif activity please refer to the FANTOM4 manuscript text and **Fig. 1** (Suzuki et al. 2009).

**ChIP-chip**

**ChIP EDGES:** The FANTOM4 consortium generated chromatin immunoprecipitation on chip data for SPI1 and SP1, for these factors, a peak of binding within 1kb of a promoter is considered positive evidence for an edge between these factors and the target gene. Public ChIP-chip and ChIP-seq datasets were also incorporated, for these we relied on the primary publication’s definition of positive binding, and we include a PubMed link back to the corresponding citation.

**ChIP WEIGHTS:** The weights used here are the number of experiments this TF has been observed binding at the target gene’s promoter (ie. a weight of 4, means it has been observed in 4 separate ChIP-chip experiments).

**miRNA**

**miRNA EDGES:** miRNA to target transcript predictions were downloaded from the EIMMO prediction server. Edges are then drawn to the corresponding Entrez Gene.

**miRNA WEIGHTS:** Weights provided are the prediction strengths from the EIMMO software.

**Perturbation**

**PERTURBATION EDGES:** siRNA knockdown(KD) of 52 transcription factors(TF) and over-expression of 12 miRNAs was used to identify TFs and miRNAs involved in THP-1 differentiation and maintenance of the undifferentiated state. Affected genes were assessed by Illumina microarrays 48 hours post transfection. The array data was quantile normalized and compared to negative control siRNAs and pre-miRNAs to identify genes that are specifically perturbed in response to the TF/miRNA.

**PERTURBATION WEIGHTS:** The perturbation edges displayed in EEDB are stringently filtered, by log fold change >=1/<=-1 and B-statistic >=2.5. A positive log FC means the transcript is induced upon knockdown of the siRNA or over-expression of the miRNA. A negative log FC means the transcript is down-regulated in response to the perturbation (eg. CD14 is strongly induced upon MYB KD and has a log FC of 6.8, whereas vitrin is strongly repressed upon MYB KD and has a log FC of -4.0).

**Other**
**OTHER EDGES:** This section contains additional nodes linked to this gene. This section includes published protein-DNA edges, and is also used to manage transcripts (accession numbers), microarray probes and Level3 promoters associated with a given gene (ie. belongs-to relationships).

For the published protein-DNA edges we include a small manuscript icon which can be clicked on and will take the user to the corresponding PubMed citation.

**OTHER WEIGHTS:** Weights for this section are set to a default of 1.

**Protein-protein interactions**

**PROTEIN-PROTEIN EDGES:** The protein-protein interactions currently displayed in EEDB are from transcription factor – transcription factor interactions only and are harvested from a number of public protein-protein interaction sources including DIP, BIND and HPRD (which is shown in the source).

**PROTEIN-PROTEIN WEIGHTS:** Weights are currently set to a default value of 1.

**PUBLISHED PROTEIN DNA**

**PUBLISHED PROTEIN DNA EDGES:** For the published protein-DNA edges we include a hyperlink to the corresponding PubMed citation.

**PUBLISHED PROTEIN DNA WEIGHT:** By default set to 1.

1. CAGE defined promoters

**CAGE DEFINED PROMOTERS**

**PROMOTER LEVELS:** For FANTOM4 we developed three levels to describe the relationship between individual transcription start sites (TSS), promoters and promoter regions. Individual TSS are referred to as level 1 (L1), nearby TSSs positions whose expression profiles are the same up to measurement noise are clustered into promoters (L2) and adjacent promoters that are within 400bp of each other are condensed into ‘promoter regions’ (L3). For further details on promoter levels please refer to the FANTOM4 main manuscript, text and **Fig. 1** (Suzuki et al. 2009).

P1^L3^ corresponds to a level 3 promoter region which can contain multiple level 2 promoters (eg. P1^L2^ and P2^L2^). Note: TFBS predictions are done per level3, BUT response weight is calculated for each level 2 promoter

1. Explanation of the subnet view

**THE SUBNET VIEW**

The subnet view allows for one to input a list of gene and/or miRNA names as nodes in a graph. The system will search for all matching connecting edges within that set of nodes based on user selectable edge filters.

**INTERFACE:**

**NODE SELECTION:** Users enter Entrez genes and mirbase microRNA IDs into the text box

**PRIMARY EDGE TYPES:** The system allows for simple logic by providing two edge sets (primary and secondary). If only edges in set1 are selected then a simple search is performed.

**SECONDARY EDGE TYPES:** If edges from both sets are selected the search is performed for genes that are connected by BOTH lines of evidence (eg. TFBS prediction in set 1 and perturbation or CHIP in set2), this can be used to prune TFBS predictions to only those for which there is some experimental support.

**EXPAND NEIGHBOURS:** Warning this is currently slow. It is used to fluff out a network one extra layer (default is off).

**HIDE SINGLETONS:** Hides nodes that have no incoming or outgoing edges (default is on)

**HIDE LEAVES:** Hides nodes with only incoming edges (default is off)

**LEGEND:**

**EDGE COLOURS:** BLACK – FANTOM4 Transcription factor binding site predictions and miRNA target predictions, YELLOW – published protein-DNA edges, PURPLE – protein-protein interactions, GREEN – chip-chip(protein-DNA) edges, RED – siRNA and miRNA perturbation edges.

**EDGE LINE STYLE**: SOLID – Direct edges, DASHED – perturbation edges (possibly direct or indirect)

**EDGE TERMINATORS:** Arrowhead – activating relationships, Blunt – repressing relationships, round – bidirectional protein-protein relationships

**NODE SHAPE:** Round nodes are genes, hexagonal nodes are miRNAs.

**NODE DIAMETER:** The diameter of each node is scaled to indicate the 'dynamics' of the gene. Calculated by mapping to log(max(detected ILMN expression)/min(detected ILMN expression)) within the time course. Highly dynamic nodes are larger than statically expressed nodes.

**NODE COLOUR:** The color of the node is mapped to a relative scale for each node between white for min(detected ILMN expression) and purple max(detected ILMN expression). If the node has no detectable ILMN expression, the name of the node becomes red and the background is white.
