# Supplementary material for: FANTOM4 EdgeExpressDB: an integrated database of promoters, genes, microRNAs, expression dynamics and regulatory interactions
Source: Genome Biol. 2009 Apr 19;10(4):R39. doi: 10.1186/gb-2009-10-4-r39 (PMC2688930; doi:10.1186/gb-2009-10-4-r39)

Additional data file 5

This supplementary note provides example views for the key monocytic marker CD14. The examples covers the gene centric and subnetwork views. The various edge types and expresison data, and how to extract the transcription factor binding site predictions from the genome browser.

Sections:

1. CAGE promoters
2. TFBS predictions
3. ChIP edges
4. Perturbation edges (siRNA against TF and miRNA overexpression)
5. Summary of inputs using sub-network view

1. CAGE promoters:

CAGE promoters are presented in 3 places within the EEDB gene centric view

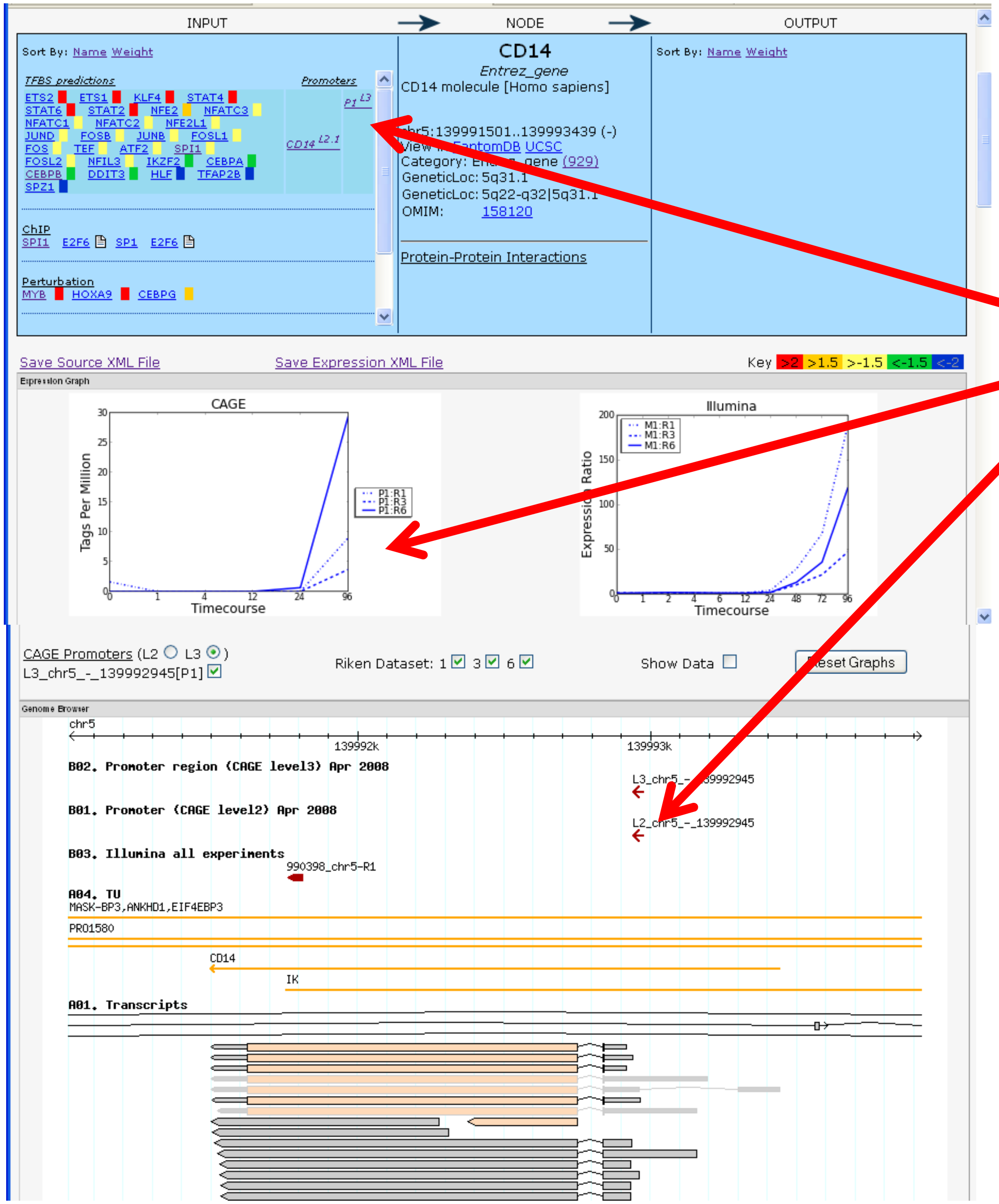

<u>CD14 promoter(L3_chr5_-_139992945)</u>

TFBS predictions (top left)

Expression (middle left)

Genomic location (bottom)

1. CAGE promoters:

Popup explaining CAGE defined promoters

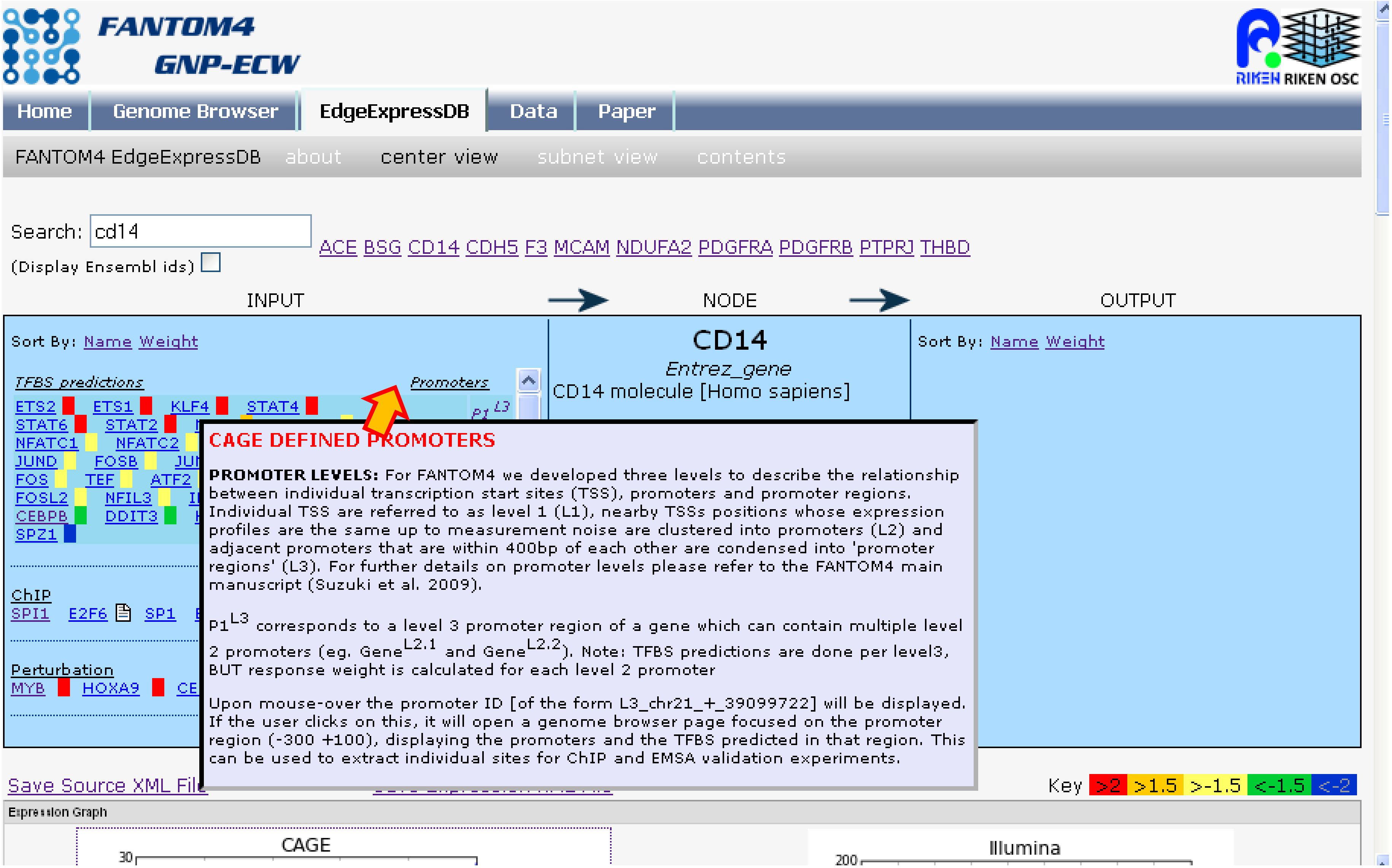

1. CAGE promoters:

For CD14, deepCAGE on THP-1 identifies one L3 promoter region (L3_chr5_-_139992945). TFBS predictions are carried out in the for a window of -300 to +100bp of each L3.

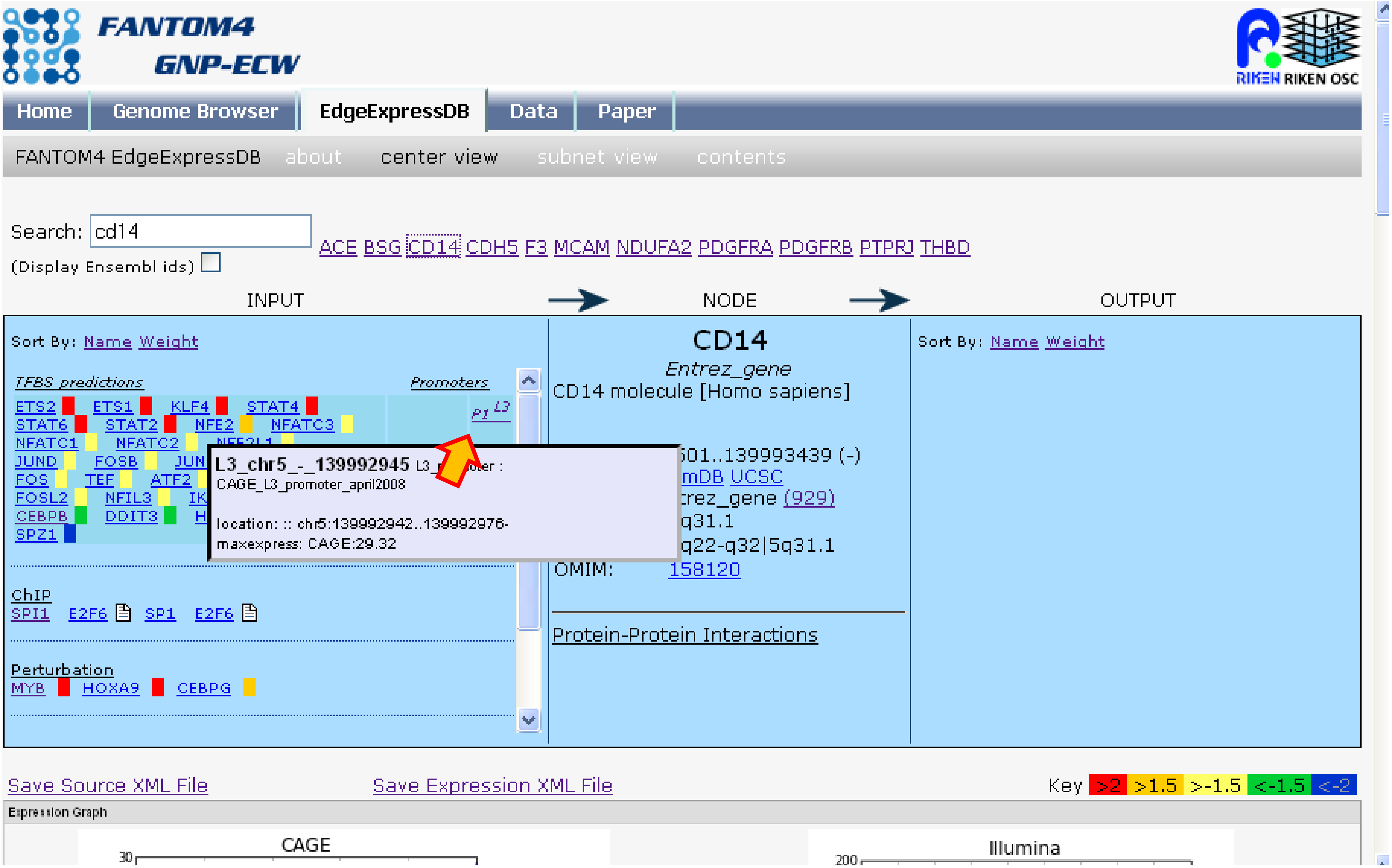

1. CAGE promoters:

The L3 promoter region (L3_chr5_-_139992945) contains one L2 promoter (L2_chr5_-_139992945).

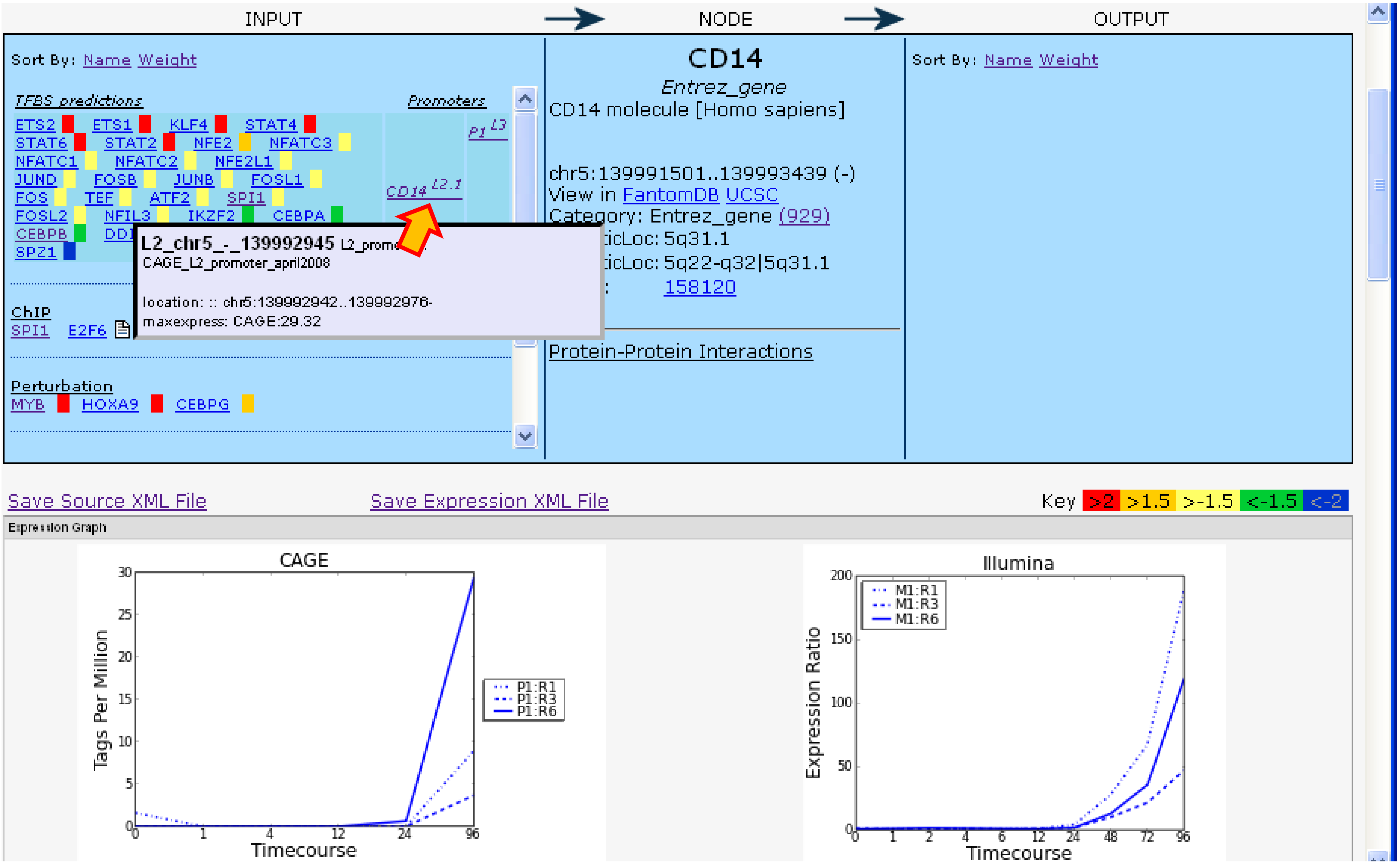

2. TFBS predictions

Popup explaining TFBS predictions and edge weights in EEDB. For each L2 promoter, response Weights are calculated that reflect how well the expression pattern of the L2, matches the

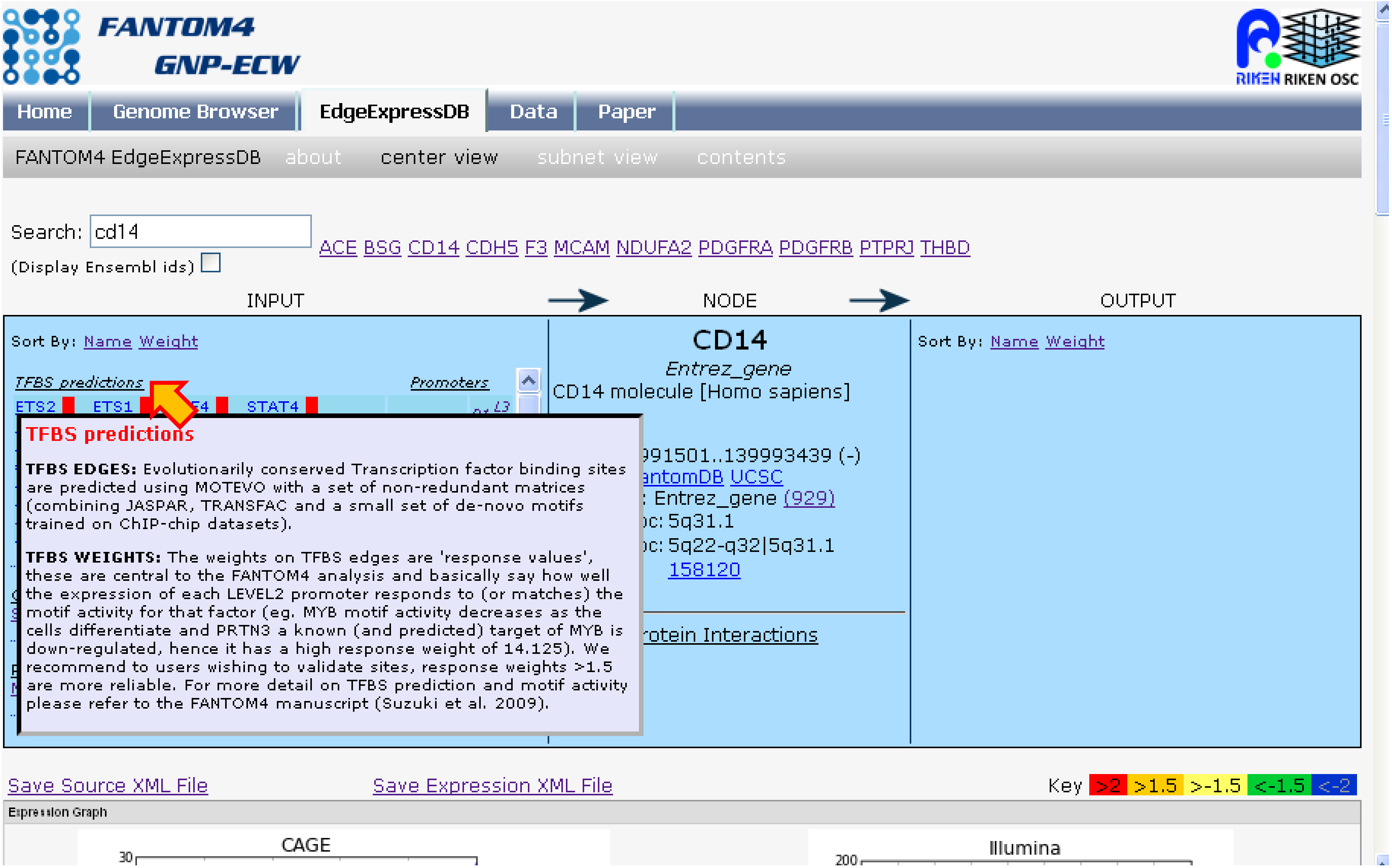

2. TFBS predictions

Popup on ETS2 – predicted regulator of CD14, weight>1.5 is high probability

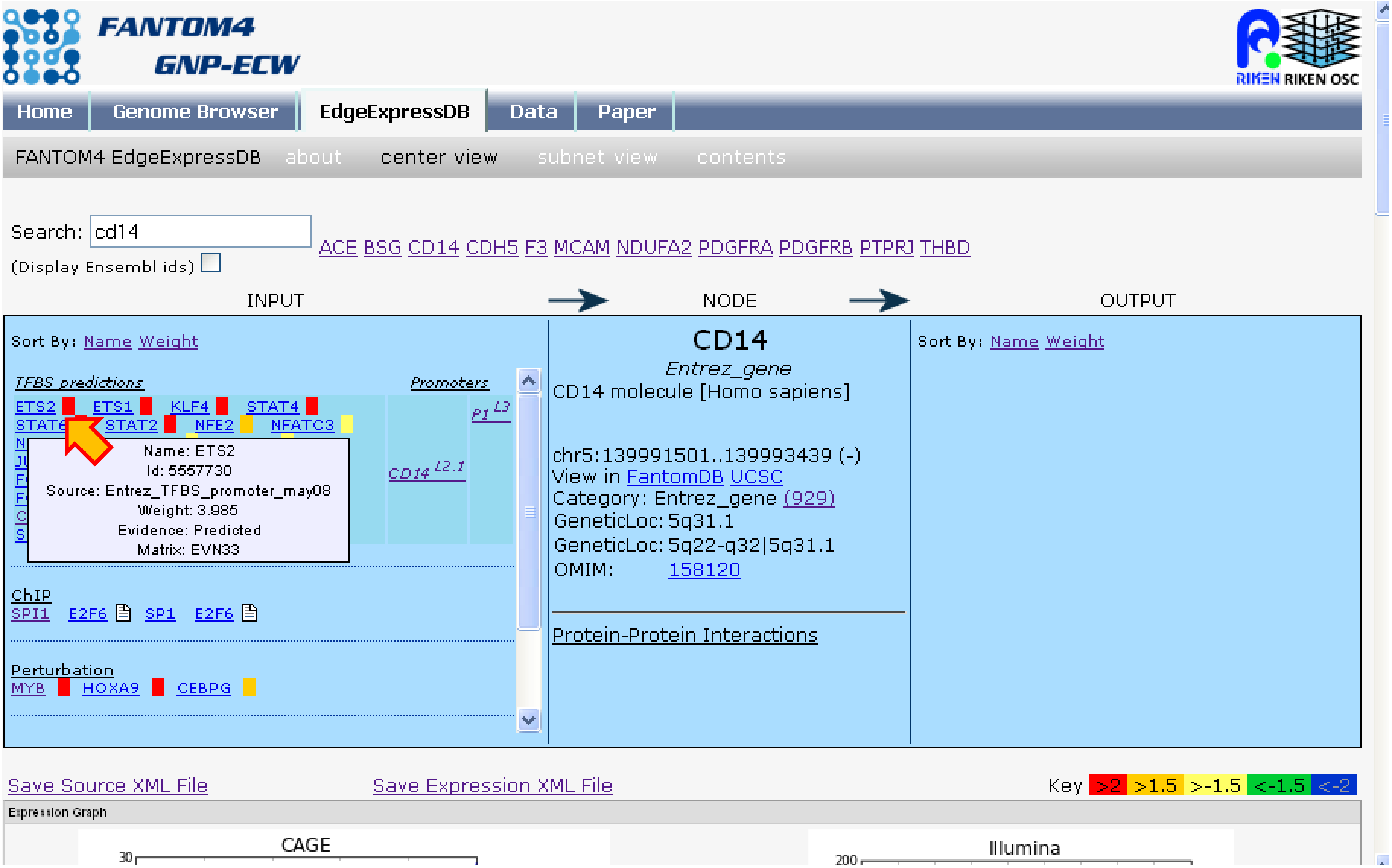

2. TFBS predictions

Clicking on either the L2 or L3 promoter will launch a genome browser window focused On the L3 promoter region showing the predicted transcription factor binding sites

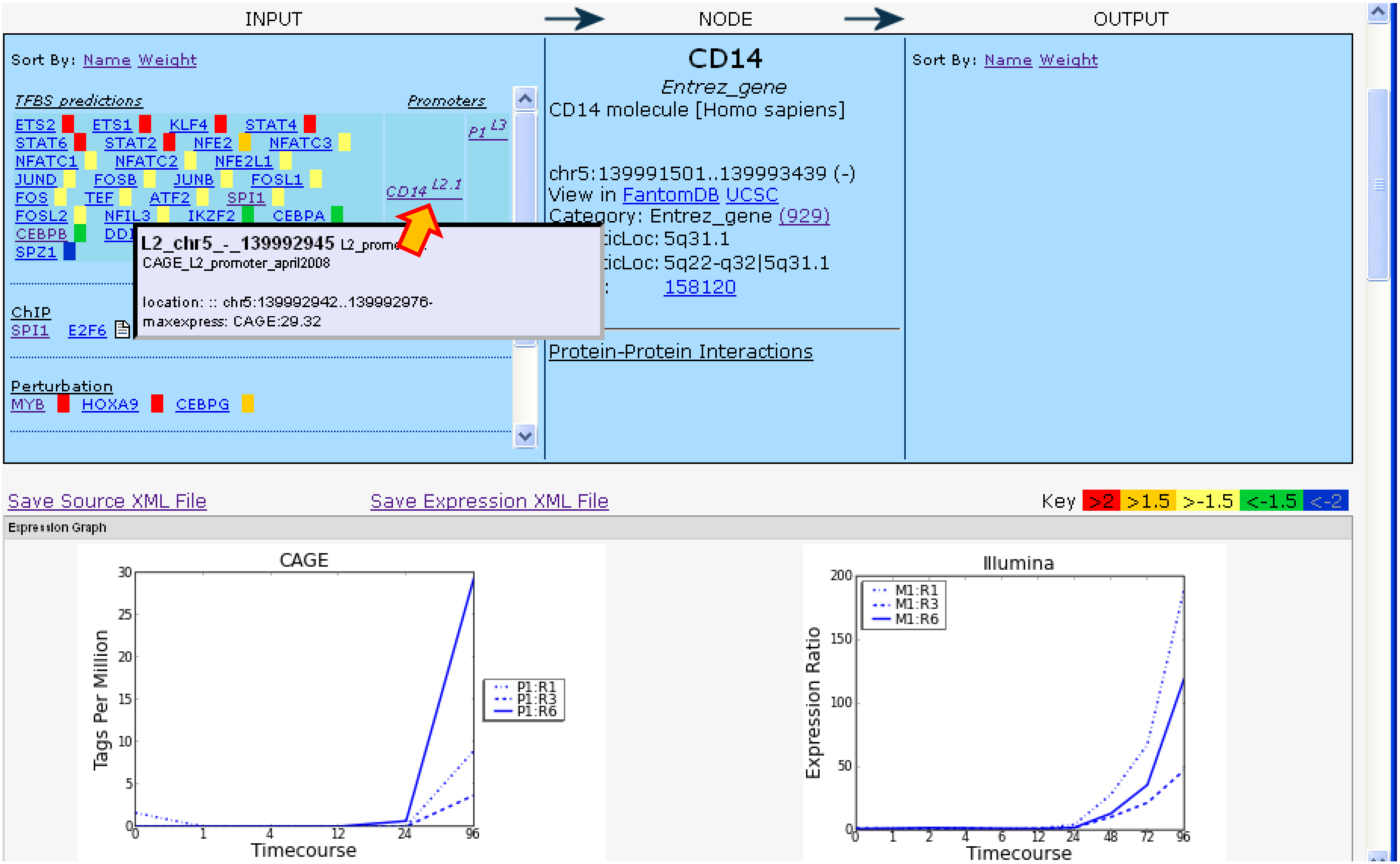

2. TFBS predictions

The window is focused on the -300, +100 bp region used for the TFBS predictions.

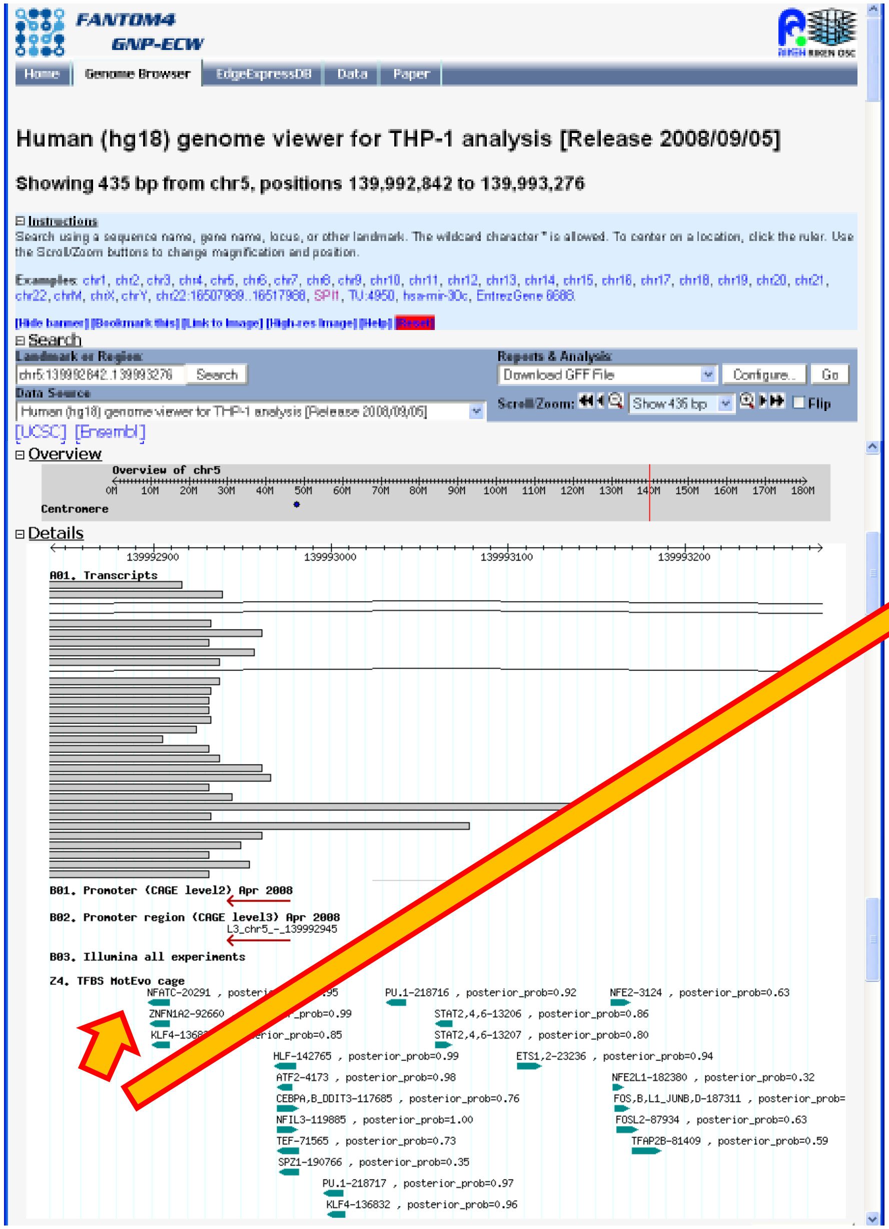

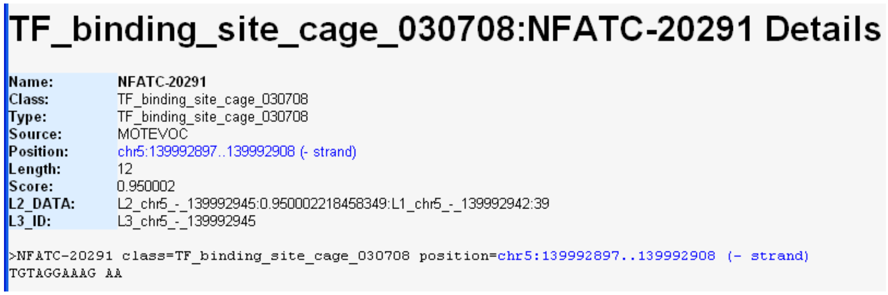

Clicking on the TFBS predictions themselves will then allow the user to get the co-ordinates Of the predicted site

3. ChIP edges

Popup explaining chromatin immunoprecipitation edges

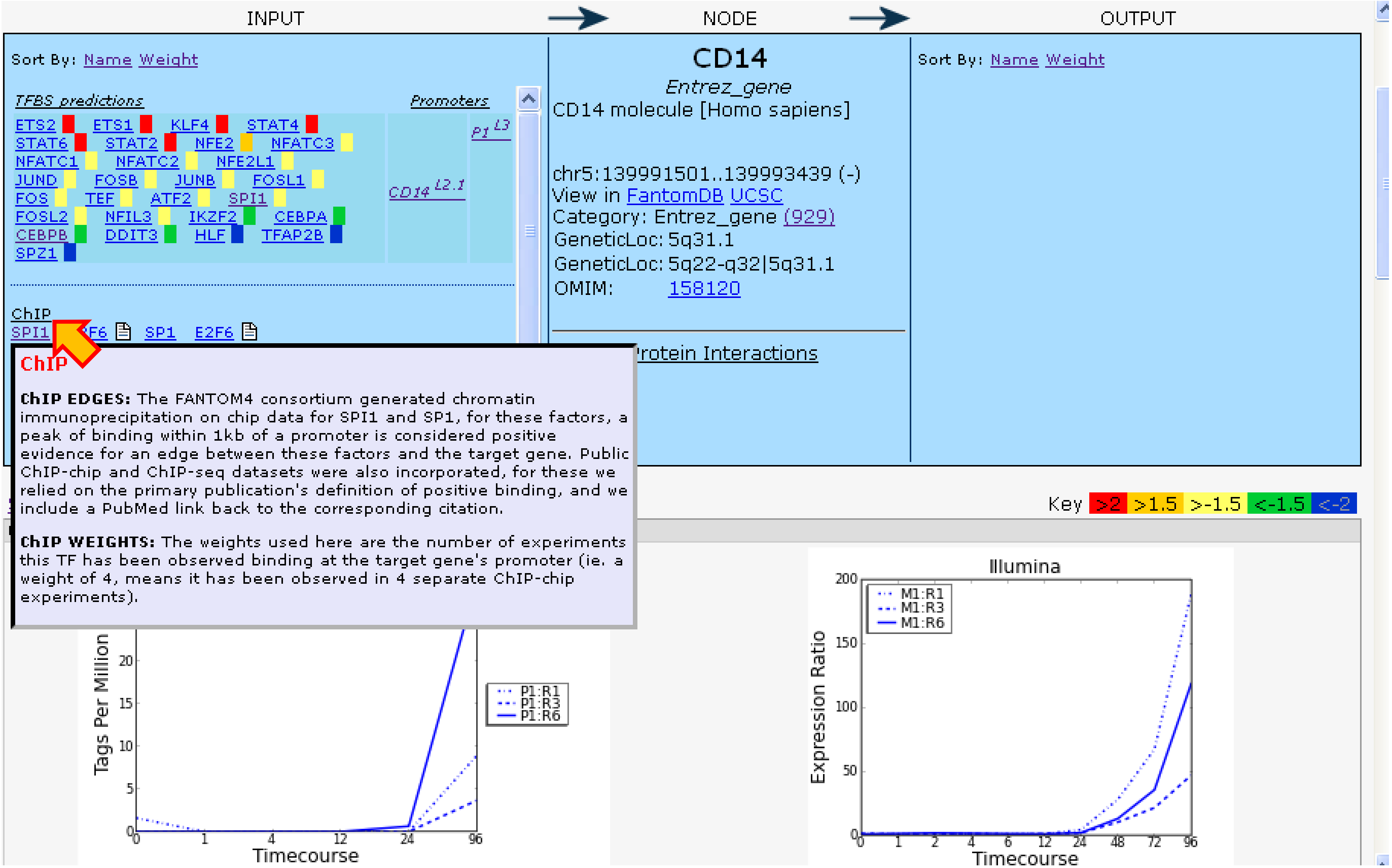

3. ChIP edges

Popup for E2F6, weight is 2, as binding was observed in 2 separate chip experiments.
Note: clicking on the manuscript icon will take the user to the pubmed article

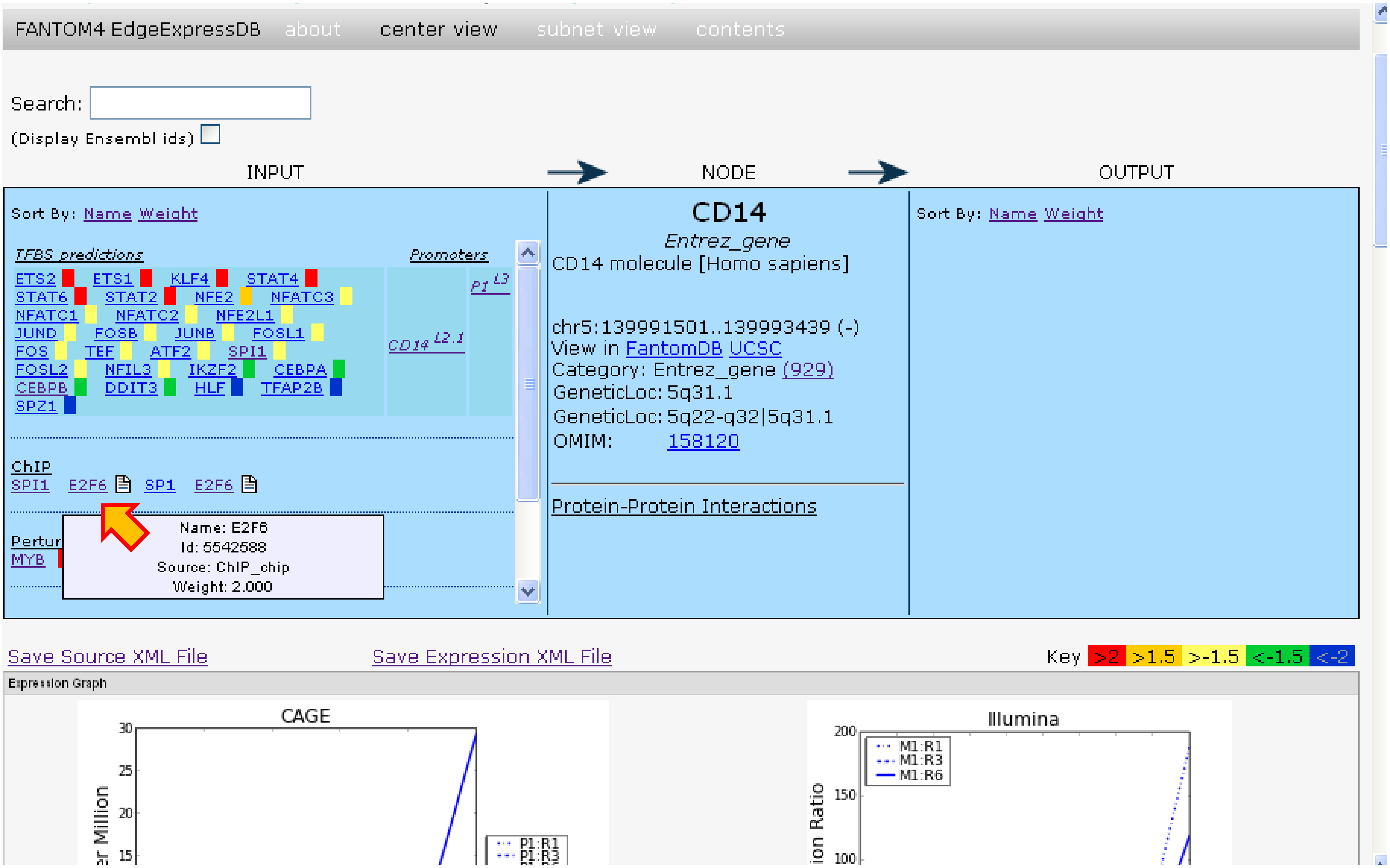

3. Perturbation edges (siRNA against TF and miRNA overexpression)

Popup explaining perturbation edges

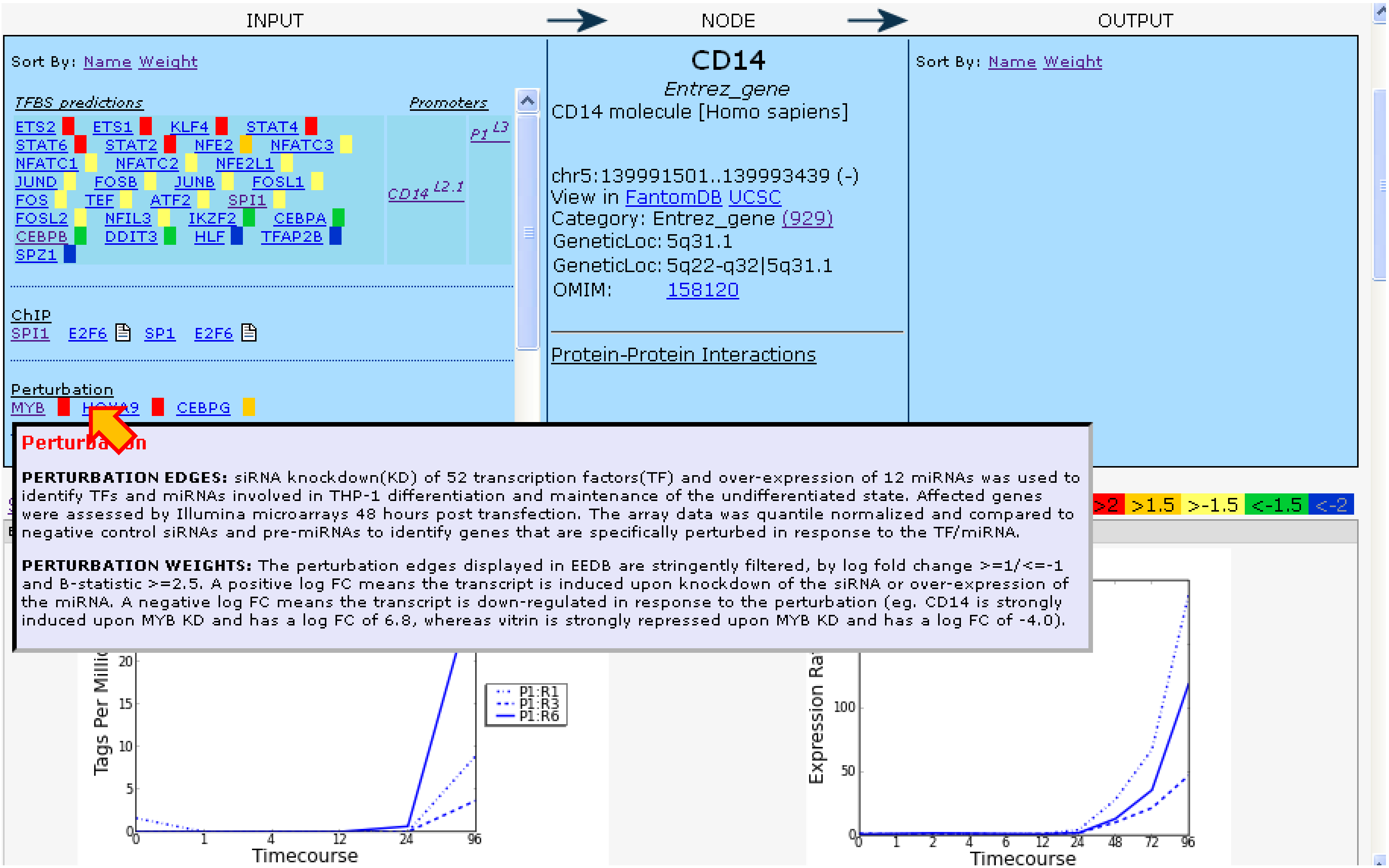

3. Perturbation edges (siRNA against TF and miRNA overexpression)

siRNA KD of MYB, HOXA9 and CEBPG induces CD14 expression in THP-1 cells as detected By Illumina microarray

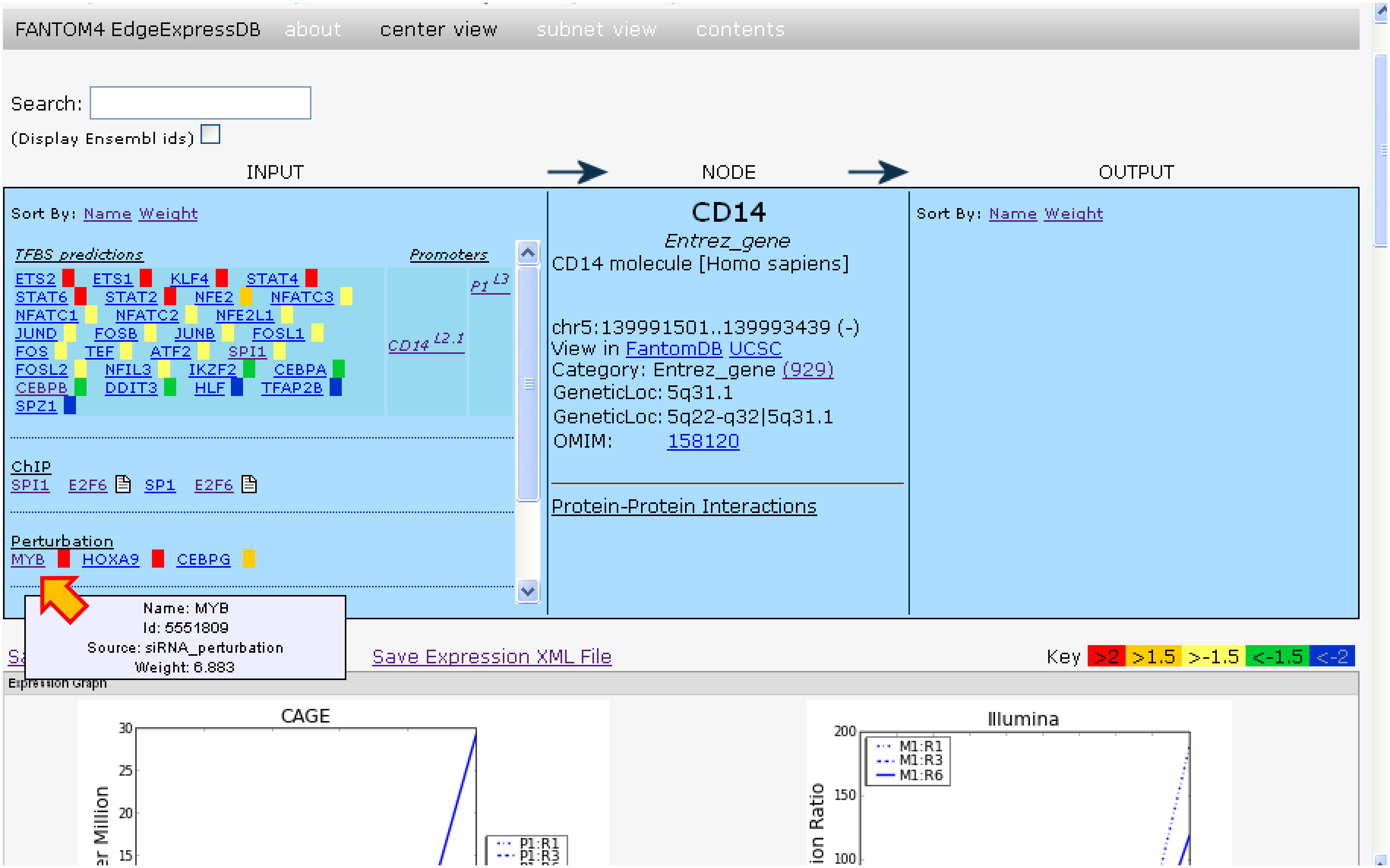

3. Summary of regulatory inputs into CD14

Pop-up tooltip explaining the interface and the legend

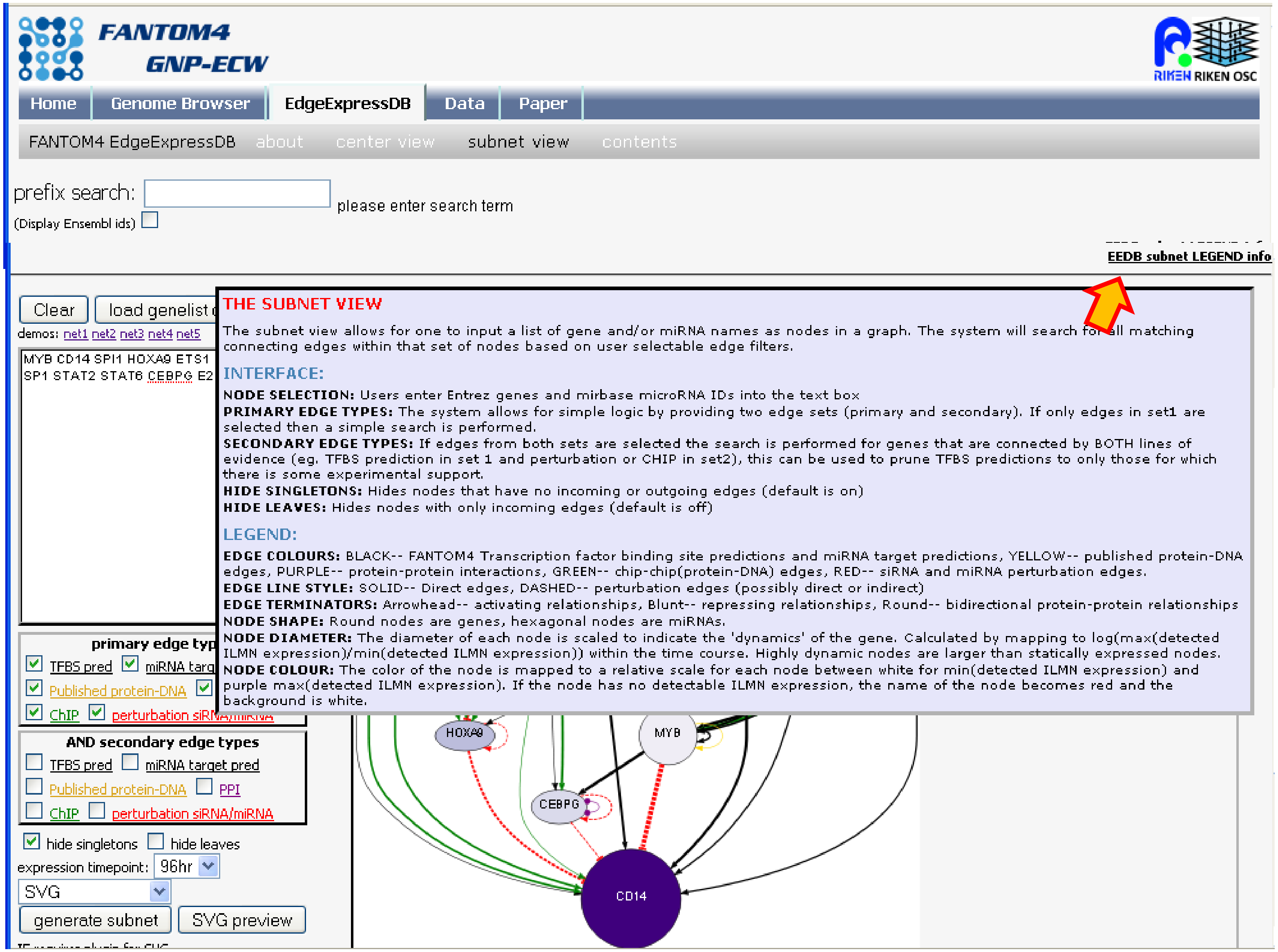

3. Summary of regulatory inputs into CD14

Sub-network view of CD14 with selected upstream regulators

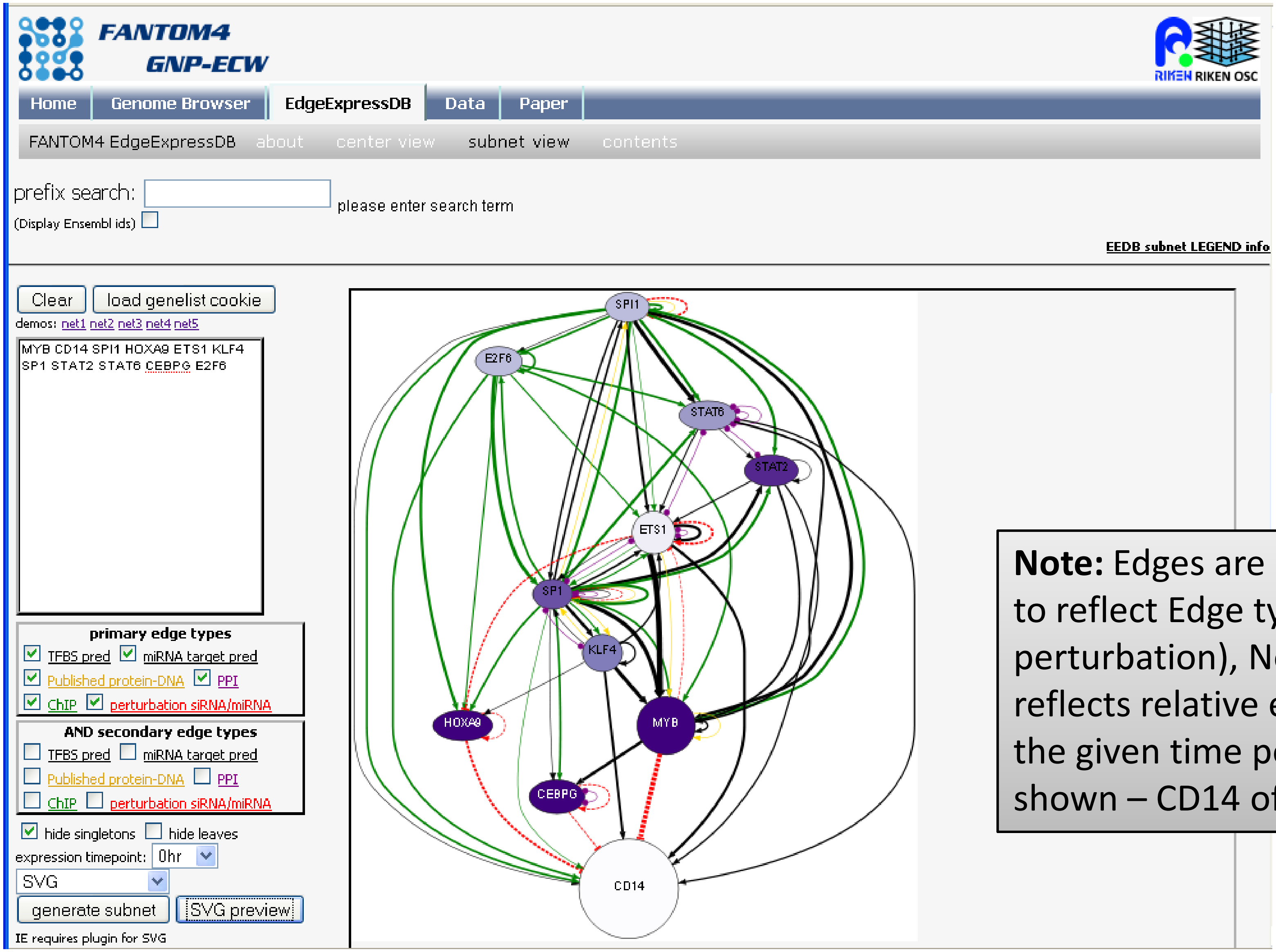

Note: Edges are colour coded to reflect Edge type (eg. red is perturbation), Node colour reflects relative expression at the given time point (0h shown – CD14 off)

3. Summary of regulatory inputs into CD14

Same sub-network view of CD14 but with 96h expression data (note CD14 is induced – purple)

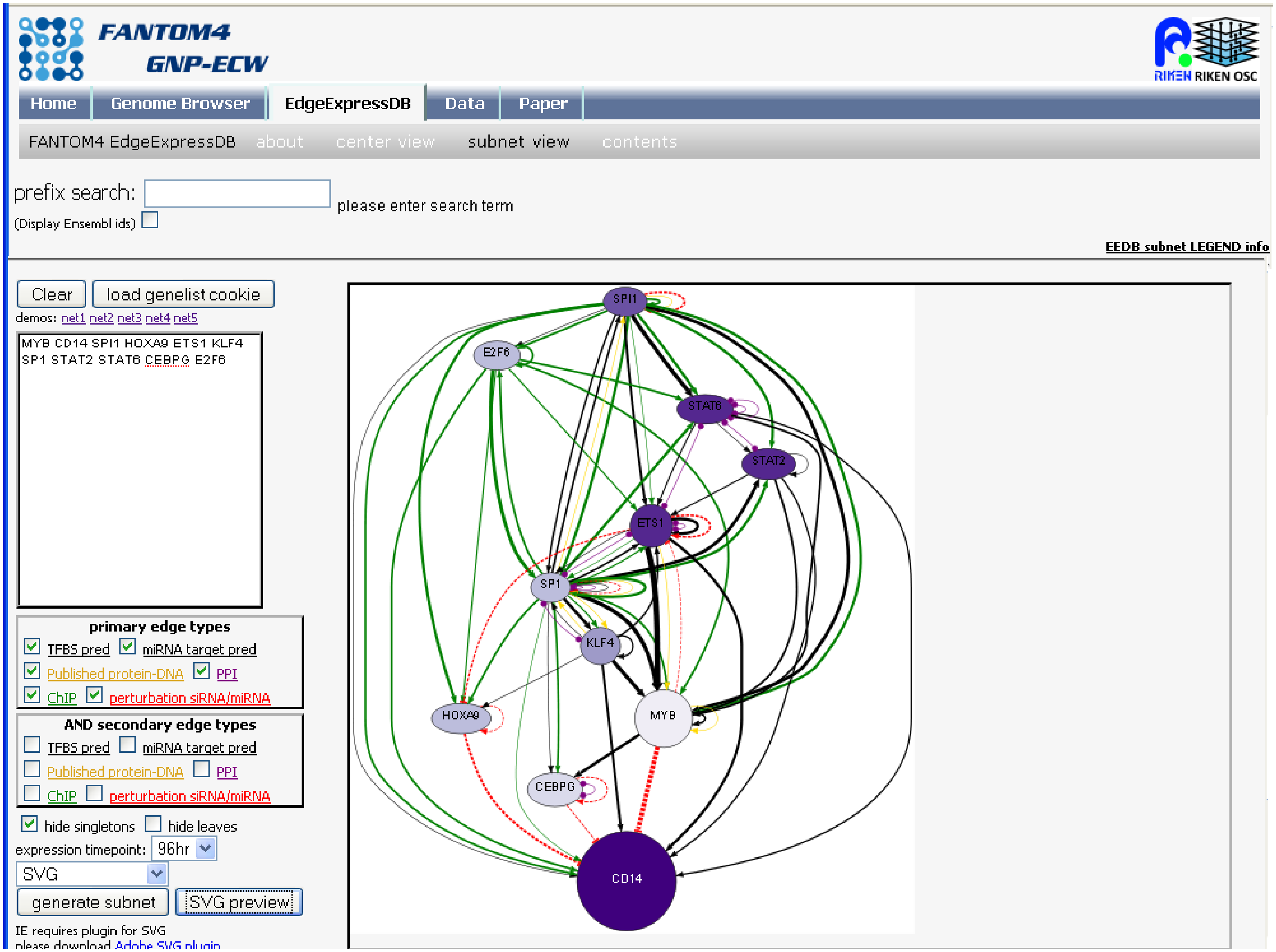

Supplement: Additional File 5 — An example of how EEDB can be used with gene-centric and sub-network views for the key monocytic marker CD14. [file gb-2009-10-4-r39-S5.pdf]
